# Molecular Characterization of the Air Microbiome and Associated Toxicological Hazards in a Zoological Garden

**DOI:** 10.3390/ijms27177839

**Published:** 2026-09-01

**Authors:** Małgorzata Okrasa, Agnieszka Maher, Tomasz Grzyb, Justyna Szulc, Adriana Nowak, Magdalena Janiszewska, Elżbieta Tarczyńska, Katarzyna Majchrzycka

**Affiliations:** 1 Department of Personal Protective Equipment, Central Institute for Labour Protection—National Research Institute, 90-133 Łódź, Poland; eltar@ciop.lodz.pl (E.T.); kamaj@ciop.lodz.pl (K.M.); 2 Department of Environmental Biotechnology, Lodz University of Technology, 90-530 Łódź, Poland; agnieszka.maher@dokt.p.lodz.pl (A.M.); tomasz.grzyb@dokt.p.lodz.pl (T.G.); adriana.nowak@p.lodz.pl (A.N.); 3 Municipal Zoological Garden in Łódź LLC., ul. Konstantynowska 8/10, 94-303 Łódź, Poland; m.janiszewska@zoo.lodz.pl

**Keywords:** zoological garden, airborne dust, bioaerosols, harmful biological agents, PPE, cytotoxicity, A-549 cell line

## Abstract

This study presents the first comprehensive characterization of the airborne microbiome in a zoological garden and its comparison with the surrounding atmospheric environment. The aim of this study was to evaluate potential human health hazards associated with occupational and visitor exposure in zoo environments across 24 sampling locations. The assessment included measurements of particulate matter (PM) and gaseous pollutants, microbiological contamination of air, surfaces, settled dust, and selected items of personal protective equipment (PPE). In addition, the cytotoxic potential of settled dust samples was evaluated using A549 human lung epithelial cells. The highest concentrations of airborne PM ranged from 0.250 mg/m^3^ for PM_1_ to 0.278 mg/m^3^ for PM_10_. The concentrations of chemical contaminants varied depending on the sampling location. Airborne bacterial counts ranged from 1.1 × 10^2^ to 3.0 × 10^2^ colony-forming units (CFU)/m^3^ in employee areas and from 2.2 × 10^3^ to 1.5 × 10^4^ CFU/m^3^ in visitor areas. The average microbial contamination of surfaces in both employee and visitor areas was 4.3 CFU/cm^2^. Amplicon sequencing revealed that the airborne microbiome was dominated by bacterial taxa belonging to Bacillota, Pseudomonadota, and Actinomycetota, as well as fungal taxa representing the phyla Ascomycota and Basidiomycota. Settled dust samples exhibited variable cytotoxicity toward A549 cells, with half-maximal inhibitory concentration (IC_50_) values ranging from 13.1 to 53.5 mg/mL. The findings provide novel insights into the molecular composition of zoo-associated airborne microbial communities and their potential implications for environmental health risk assessment and hygiene management practices in zoological gardens.

## 1. Introduction

Zoological gardens, in the form of collections of animals for display, were already known in Egyptian, Roman, and Chinese civilizations more than 4500 years ago. Then, the concept of animal shows in cages developed in the 13th century. The world’s first zoological park was founded in Vienna in 1751 (Tiergarten Schönbrunn) and has been operating continuously to this day. In the 1990s, zoos aroused great public interest, and there were already more than 10,000 such facilities around the world [1,2].

Today, zoological gardens are attractive places for entertainment, but they also play an invaluable role in wildlife conservation. However, the stricter definition applied to institutions meeting internationally recognized standards for animal welfare, education, and conservation comprises approximately 1500 zoos worldwide [3]. Of these, nearly 400 are members of the World Association of Zoos and Aquariums (WAZA) and meet the guidelines of this institution for animal care and welfare, environmental education, and global conservation [3].

Air quality in a zoological garden environment is very complex due to the coexistence of diverse animal species in relatively small areas shared with animal keepers, other employees, and visitors. Among the key factors shaping this environment is particulate matter (PM), which serves as a carrier of both chemical and biological contaminants and is therefore of particular concern for human exposure. PM in dust is commonly classified by aerodynamic diameter (AD) into PM_10_ (<10 µm), PM_2.5_ (<2.5 µm), respirable particles (<4 µm), and submicron particles PM_1_ (<1 µm) [4]. Particle size determines deposition within the respiratory tract: larger particles (5–10 µm) remain in the upper airways, smaller ones (1–5 µm) reach the tracheobronchial region, and the finest (<1 µm) penetrate into the alveoli [5]. Chemically, PM consists of inorganic ions, carbonaceous compounds, and various metals and metalloids [6,7]. Secondary inorganic species such as nitrate (NO_3_^−^), sulfate (SO_4_^2−^), and ammonium (NH_4_^+^) are also present in a significant portion of PM [8,9]. The thousands of organic compounds of organic carbon (OC) are natural parts of dust, while elemental carbon (EC) is emitted directly from combustion processes [10].

From a microbiological perspective, the composition of dust includes bacteria, fungi, viruses, pollen, plant debris, and their byproducts (endotoxins, allergens, and mycotoxins) [11]. The following microorganisms were detected in animals or humans exposed to animals from a variety of settings (e.g., zoo, farms, and veterinary clinics), as well as directly from the environments these animals inhabit: *Shigella flexneri* in monkeys [12]; *Streptococcus agalactiae* in elephants [13]; *Staphylococcus succinus* and *Staphylococcus vitulinus* from the inhabitant of giraffes, elephants, camels, monkeys, and kangaroos [14]; *Staphylococcus haemolyticus* from the inhabitant of monkeys [14]; *Acinetobacter* and *Pseudomonas* in pig farms, and *Staphylococcus*, *Pseudomonas*, and *Corynebacterium*, which were dominant bacterial genera in pig farmers [15]; *Corynebacterium*, *Acinetobacter*, *Staphylococcus*, *Sphingomonas*, *Flavobacterium*, *Cladosporium*, *Alternaria*, *Aspergillus*, *Penicillium*, *Trichosporon*, *Verticillium*, and *Talaromycesn* in scattered farms [16]; *Fusarium* and *Cladosporium* in horse stables [17]; *Penicillum* and *Aspergillus* in horse stables and veterinary clinics [17,18]; Multidrug-Resistant Methicillin-Resistant *Staphylococcus aureus* (MRSA) in chicken farms [19]; and *Escherichia coli* O157:H7 in goats [20]. In accordance with Directive 2000/54/EC [21], *Escherichia coli* verocytotoxigenic strains (e.g., O157:H7 or O103) and *Shigella dysenteriae* (Type1) were classified in the third group of biological agents (can cause severe human disease and present a serious hazard to workers; they may present a risk of spreading to the community, but there is usually effective prophylaxis or treatment available), while *Pseudomonas aeruginosa*, *Shigella flexneri*, *Staphylococcus aureus*, and *Streptococcus* spp. were classified in the second group of biological agents (can cause human disease and might be a hazard to workers but it is unlikely to spread to the community; there is usually effective prophylaxis or treatment available). Epidemiological research has indicated that poor air quality can adversely affect human health and contribute to morbidity and mortality, as well as cause cardiovascular and respiratory diseases [22,23,24,25]. As people spend up to 80% of their time indoors, the quality of indoor air is a critical determinant of human health. Consequently, Sick Building Syndrome (SBS) has been strongly linked to indoor air pollution, particularly exposure to PM, carbon dioxide (CO_2_), ozone (O_3_), nitrogen dioxide (NO_2_), sulfur dioxide (SO_2_), and a wide range of volatile organic compounds (VOCs) [26,27,28,29]. In cytotoxicity testing, the phenomenon of PM inducing cytotoxic effects on the A-549 cell line is strongly correlated with PM size [4]. Accordingly, another group of researchers observed elevated expression levels of inflammatory agents, such as *TNFα*, *IL6*, and *CYP1A1* genes, in mammalian cell lines after treatment with dust samples [7].

Considering the wide range of air constituents, including contaminants, research on air quality in zoological gardens is very limited. Only a few publications were identified regarding airborne microbiological contamination in zoos, which are sequential extension of the authors’ previous research [14,30,31,32]. The above-mentioned studies provide evidence for the urgent need to characterize air pollution elements (e.g., dust, chemicals, and bioaerosols) to ensure the health of animal workers and animals. Building on this gap, it becomes evident that not only the characterization of airborne contaminants but also the assessment of protective measures against them requires more attention. Research on protection against air pollutants in zoo environments is still lacking, and no data were found in this context. The inclusion of protective goggles in the present study was therefore warranted, as these devices are already used by zoo workers as part of their routine personal protective equipment (PPE). Given that zoo environments often generate airborne dust, bioaerosols, and allergenic particles that may contribute to eye irritation or discomfort, it was important to assess not only the contamination of workplace surfaces but also that of the goggles themselves, which represent a commonly handled and repeatedly reused item of equipment. Evidence from healthcare settings shows that protective goggles and face shields can reduce allergic conjunctivitis, dry eye, and ocular surface dysfunction linked to aerosolized microorganisms and PM [33]. Although ocular outcomes have not yet been systematically examined in zoological facilities, environmental sampling has demonstrated significant levels of potential allergens and microbial agents [31]. Another important factor is that goggles in such settings are typically stored loosely at workplaces, making them prone to microbial accumulation on their surfaces and thereby increasing the risk of ocular exposure [33]. PM in indoor environments represents an important exposure factor with documented adverse health effects [34,35]. It should also be emphasized that the eyes of animal workers are exposed not only to airborne contaminants but also to pathogens and allergens transmitted through direct contact—for example, when touching the face or eyes with contaminated hands after interacting with animals. For these reasons, the present study investigated not only the contamination of workplace surfaces but also protective goggles.

The aim of this study was to characterize air quality, airborne microbial communities, environmental contamination, and the cytotoxic potential of settled dust in selected areas of a zoological garden and to identify factors potentially relevant to human exposure.

This study included dust, gaseous pollutants, and microbiological contamination of air and surfaces, as well as selected items of PPE. In addition, this is the first study to determine the composition of the air microbiome in a zoo compared to the air surrounding the facility. Moreover, the research utilized cytotoxicity testing on the human adenocarcinoma lung (alveolar) epithelial adherent cell line A-549 to assess potential health hazards associated with components of settled dust. To date, such an extensive analysis of physicochemical, biological, and toxicological hazards in zoos has not been conducted.

The present study was designed as an environmental characterization of zoo-associated microbial and physicochemical contamination rather than a quantitative assessment of personal exposure.

## 2. Results and Discussion

### 2.1. Microclimatic Conditions

The microclimatic assessment across the sampling locations showed relatively stable thermal conditions (Table S1), with most sites maintaining temperatures between 20 and 26 °C, except for the cold room (E7) at around 12 °C. Relative humidity ranged widely, from approximately 23% in transitional corridors (E9–E10) to over 80% in the aquatic area (V1), reflecting functional differences between spaces. Airflow velocities were generally low (<0.1 m/s) in enclosed rooms such as veterinary and storage areas, while higher values (0.1–0.3 m/s) were observed in animal facilities and aquatic sections, consistent with greater ventilation requirements.

When location-level mean values were compared between employee and visitor areas using the Kruskal–Wallis test, no statistically significant difference in temperature was found between the two zones (22.1 ± 3.7 vs. 23.5 ± 2.2 °C; *p* = 0.273). In contrast, relative humidity was significantly higher in visitor areas than in employee areas (59.2 ± 10.1% vs. 32.2 ± 5.8%; *p* < 0.001). Airflow velocity was also significantly higher in visitor areas (0.124 ± 0.071 m/s) than in employee areas (0.052 ± 0.053 m/s; *p* = 0.016). These findings indicate that the two zones differed primarily in humidity and airflow conditions rather than temperature.

### 2.2. Airborne PM Concentrations

**Figure 1:**
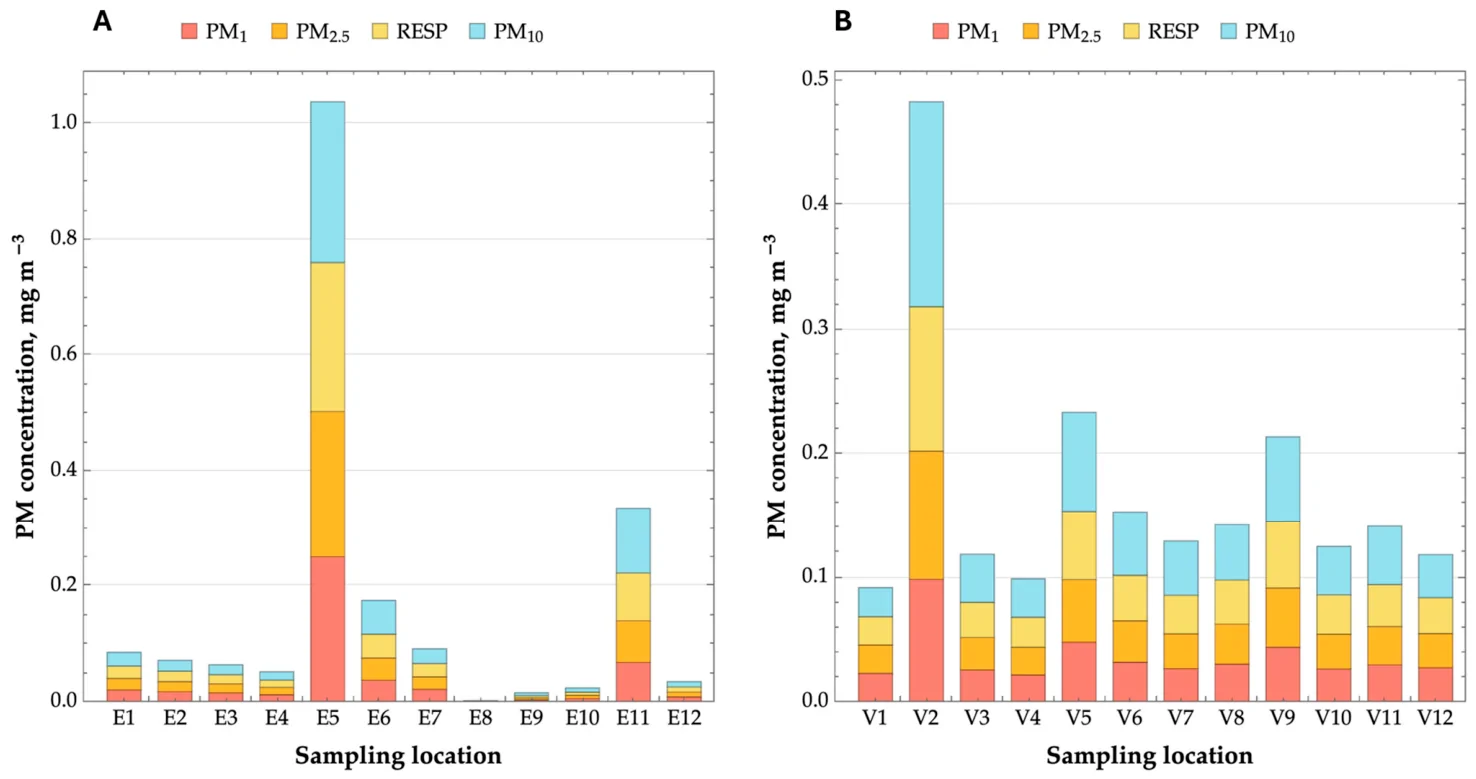
Airborne dust concentrations; (**A**) employees’ area and (**B**) visitors’ area. Particulate matter (PM); respirable fraction (RESP).

Short-term measurements of airborne PM concentrations performed in twelve employees’ areas (E1–E12, Figure 1A) and twelve visitors’ areas (V1–V12, Figure 1B) showed variability among the sampled locations within both zones. The analysis was conducted for four particle fractions: PM_1_, PM_2.5_, the respirable fraction (RESP), and PM_10_. These values represent short-term concentrations recorded during the individual sampling periods and should not be interpreted as time-weighted occupational or daily environmental exposure levels.

In the employees’ areas, the highest concentrations were observed at location E5, where mean values ranged from 0.250 mg/m^3^ for PM_1_ to 0.278 mg/m^3^ for PM_10_, accompanied by considerable variability. A second hotspot was identified in E11, with mean concentrations between 0.067 mg/m^3^ and 0.113 mg/m^3^, depending on the fraction. In contrast, most of the other employees’ locations exhibited substantially lower levels, ranging from values close to the detection limit in E8 up to 0.058 mg/m^3^ for PM_10_ in E6. Several areas, such as E2, E3, E7, and E12, showed relatively uniform and low concentrations, not exceeding 0.03 mg/m^3^ for PM_10_.

In the visitors’ areas, the highest levels were recorded in V2, where average concentrations ranged from 0.098 mg/m^3^ (PM_1_) to 0.165 mg/m^3^ (PM_10_). Elevated dust loads were also detected in V5 and V9, with PM_10_ concentrations of 0.080 and 0.068 mg/m^3^, respectively. Most visitors’ sites, however, were characterized by moderate concentrations, with typical values in the ranges 0.02–0.05 mg/m^3^ for PM_1_–RESP and 0.03–0.05 mg/m^3^ for PM_10_. The lowest concentrations were measured in V1 and V4, where mean PM_10_ values did not exceed 0.031 mg/m^3^.

To compare the two zones, the mean concentration obtained for each sampling location was treated as one independent observation. Location-level concentrations of PM_1_, PM_2.5_, RESP, and PM_10_ were compared between employee and visitor areas using the Kruskal–Wallis test. Statistically significant differences between the two zones were found for all four particulate-matter fractions: PM_1_ (*p* = 0.028), PM_2.5_ (*p* = 0.028), RESP (*p* = 0.028), and PM_10_ (*p* = 0.028). Median location-level concentrations were higher in visitor areas than in employee areas for each fraction, indicating that elevated PM concentrations were more consistently observed across visitor-accessible locations. The arithmetic means for PM_1_ and PM_2.5_ in the employee zone were strongly influenced by the exceptionally high concentrations recorded at E5.

To further characterize the particle-size distribution, the coarse aerosol fraction was calculated by subtracting PM_1_ from PM_10_ for each sampling location (Table S3). Mean coarse-particle concentrations ranged from 0 mg/m^3^ at E8 to 0.067 mg/m^3^ at V2. The highest coarse-particle concentrations were observed at V2 (0.067 mg/m^3^), E11 (0.046 mg/m^3^), V5 (0.032 mg/m^3^), E5 (0.028 mg/m^3^), and V9 (0.025 mg/m^3^). Depending on the sampling location, the calculated coarse fraction accounted for 0–47.6% of the measured PM_10_ concentration. Contributions of 30–40% or more were observed at several locations, including E6, E9, E11, and multiple visitor-accessible sites (V2–V11), indicating that particles exceeding 1 μm significantly add to the local PM_10_ load.

Coarse particles are predominantly associated with mechanical generation processes, such as the resuspension of previously deposited dust, rather than with gas-to-particle conversion or high-temperature combustion. In zoological environments, this may result from the movement of animals, employees and visitors, cleaning activities, handling of feed and bedding materials, or disturbance of deposited dust from floors and other surfaces. The substantial contribution of the coarse aerosol fraction, observed at several sampling locations, is consistent with a significant role of local resuspension in airborne PM. However, in our studies, the coarse fraction did not constitute the majority of PM_10_ at all sampling locations, and no direct source-apportionment analysis was performed. Consequently, the present results support resuspension as a plausible and potentially important local source rather than establishing it as the dominant source of PM. Nevertheless, measures aimed at limiting the accumulation and mechanical disturbance of settled dust may be implemented to reduce coarse-particle concentrations.

The above results are consistent with previous studies, which have shown that airborne dust concentration in zoo premises is relatively low (0.048–0.204 mg/m^3^), with the highest values observed for the PM_10_ fraction, intermediate for PM_4_ and PM_2.5_, and the lowest for PM_1_ and total PM concentrations [30,31].

The measured PM concentrations should be interpreted as short-term, location-specific measurements obtained during 3 min sampling periods. As the individual locations were monitored sequentially rather than simultaneously, the observed between-location differences may partly reflect temporal variation in occupancy, animal and staff activity, feeding or cleaning operations, door opening, ventilation conditions, and other transient local sources. Therefore, the results characterize the conditions prevailing at each location at the time of measurement and allow the identification of areas with relatively elevated short-term PM concentrations. These measurements do not represent personal exposure or 8 h time-weighted occupational exposure, nor are they equivalent to the 24 h averages used for assessing ambient-air PM_10_ concentrations. Consequently, they cannot be used to demonstrate compliance or non-compliance with occupational or ambient-air limit values. Similarly, the recorded concentrations cannot be classified as safe or hazardous based on short-term measurements alone.

It is worth noting that PM in animal housing contains solid and liquid biological matter comprising feed particles, dander, animal excretions, viruses, and microorganisms [36]. Exposure to excessive PM concentrations increases the risk of cardiovascular disease, perinatal complications, and neurological and cognitive disorders [37]. Furthermore, workers can transfer dust particles on their shoes and clothing from animal housing to residential buildings, thus posing a risk to others. Therefore, the use of personal protective equipment (half-masks, eyewear, clothing, and footwear) in such workplaces may be necessary.

### 2.3. Airborne Chemical Contaminant Levels

**Figure 2:**
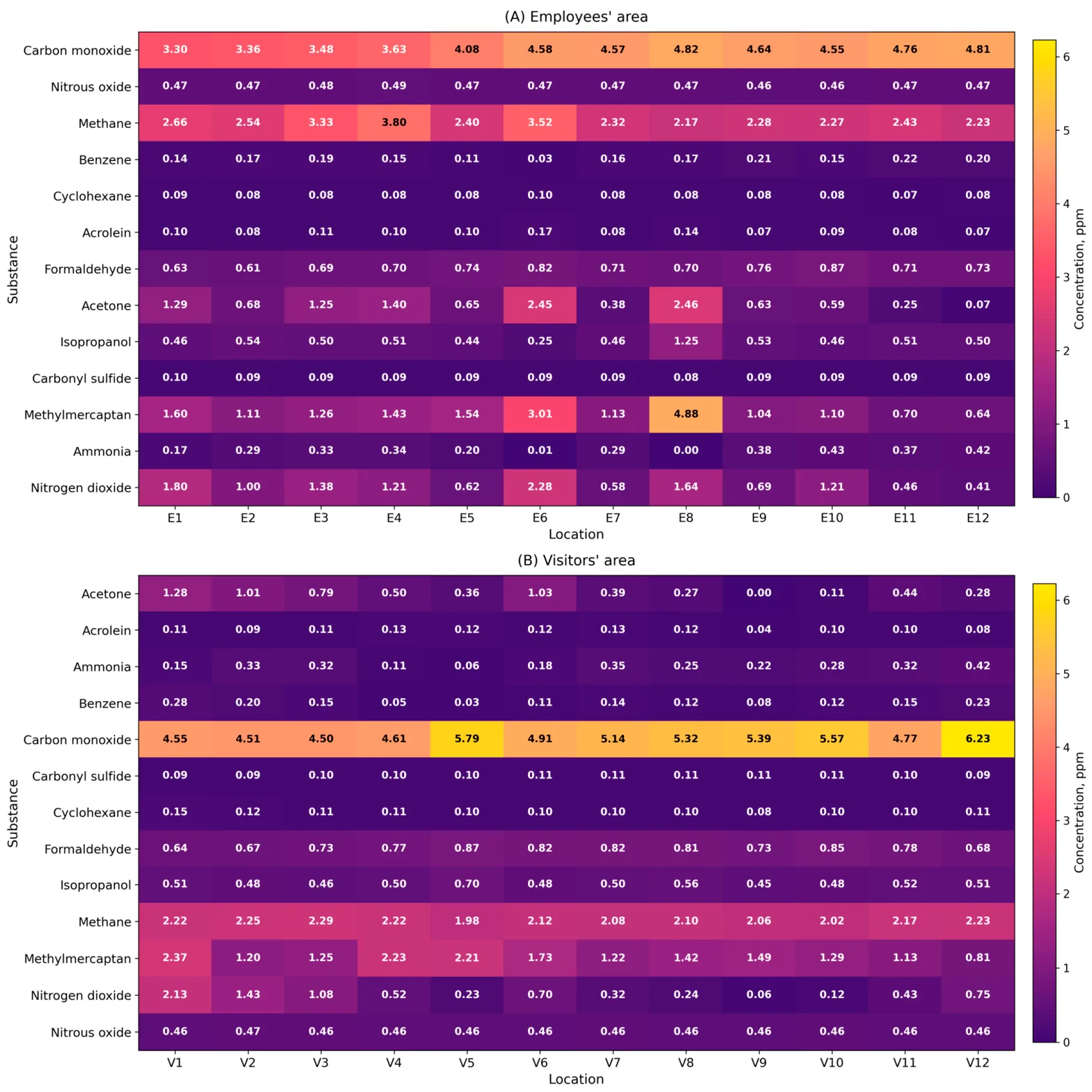
Spatial distribution of measured gas concentrations in different functional areas of the zoo (mean values, ppm); (**A**) employees’ area and (**B**) visitors’ area.

Air quality monitoring in twelve employees’ areas revealed emission patterns reflecting the specific functions of each space (Figure 2A). Veterinary and administrative rooms (E1, E2, and E8) showed elevated formaldehyde, acetone, and isopropanol, consistent with cleaning and disinfectant use, while the veterinary bird cage (E2) also contained ammonia and mercaptans from animal waste. Staff facilities presented mixed profiles: the kitchen (E3) contained methane and volatile organics from food preparation, whereas the cloakroom (E4) carried residual biological gases likely transferred on clothing. The workshop (E5) was distinguished by combustion-related pollutants (CO and NO_2_) alongside solvent-like compounds. The highest biological emissions occurred in food storage and cold rooms (E6–E7), where methane, ammonia, mercaptans, and acetone indicated decomposition and fermentation of organic matter. Animal areas such as the elephant (E10) and monkey (E12) headquarters displayed strong methane and ammonia signals, with additional CO and NO_2_ in the elephant building linked to heating or vehicles.

Visitor-accessible areas showed similarly distinct profiles shaped by enclosure type and ventilation (Figure 2B). The reference locations (V1 and V12) contained intermediate concentrations of CO, methane, and NO_2_, while the visitors’ cloakroom (V2) showed acetone and isopropanol, suggesting contamination from clothing or disinfectant residues. Biological gases dominated in animal enclosures, including methane and mercaptans in the elephants’ (V3, V9, and V10) and macaques’ zones (V4 and V8). The macaques’ first floor additionally contained acetone and isopropanol, while the elephant bathing area (V10) had methane, ammonia, and isopropanol from combined biological and water treatment processes. Elevated CO and volatile organics were recorded in the oceanarium main square (V5), and the tunnel (V11) displayed a broader spectrum of NO_2_, mercaptans, and ammonia, reflecting limited ventilation and accumulation of emissions.

The overall gaseous-pollutant profiles differed significantly between employee and visitor areas, as demonstrated by statistical analysis performed for location-level mean concentrations (*p* = 0.0001). The homogeneity of multivariate dispersion assumption was met (*p* = 0.349), indicating that the observed difference was not attributable to unequal within-zone variability. Compound-specific comparisons were performed using the Kruskal–Wallis test. Carbon monoxide, cyclohexane, and carbonyl sulfide concentrations were significantly higher in visitor areas, whereas methane and nitrous oxide concentrations were significantly higher in employee areas (*p* < 0.05). No statistically significant differences between the zones were found for benzene, acrolein, formaldehyde, acetone, isopropanol, methylmercaptan, ammonia, or nitrogen dioxide. These results indicate that the two zones differed in their overall gaseous-pollutant profiles, although statistically significant zone-related differences were limited to selected compounds.

Across both staff and visitors’ areas, three dominant patterns were observed: (i) biological emissions (methane, ammonia, and mercaptans) in food-related and animal areas; (ii) combustion-related pollutants (CO and NO_2_) in technical facilities and semi-enclosed visitor spaces; and (iii) disinfectant- and solvent-related volatiles in veterinary, administrative, and transitional areas. Our findings highlight priority interventions, including improved ventilation in storage and animal enclosures, reduction in combustion emissions near visitor routes, and careful management of chemical use to limit occupational and public exposure.

### 2.4. Microbial Contamination in the Zoo Environment

#### 2.4.1. Airborne Microbial Contamination

**Figure 3:**
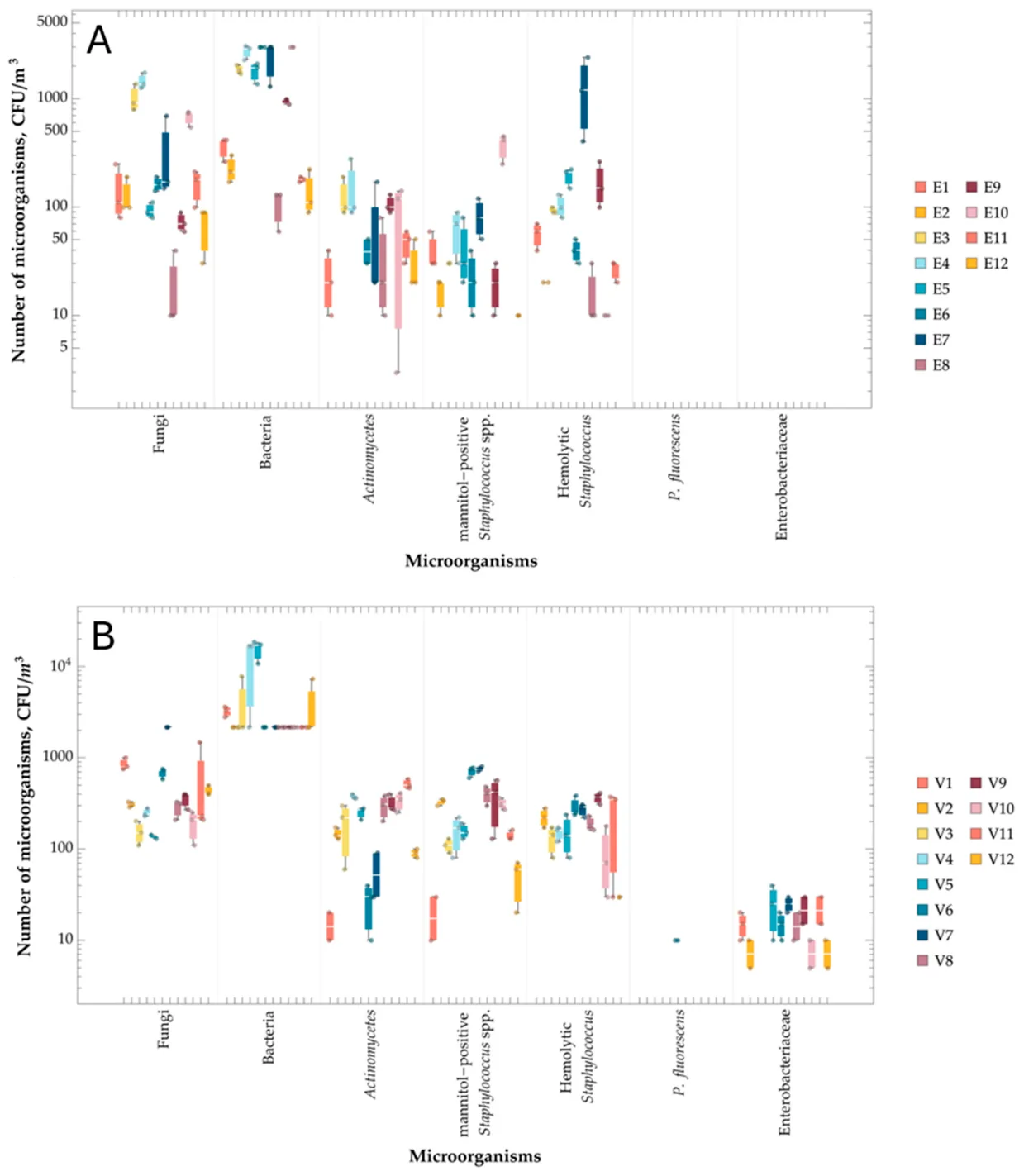
Boxplot showing median levels of microbiological contamination in the (**A**) employees’ area (E1–E12 air sampling sites); (**B**) the visitors’ area (V1–V12 air sampling sites). The ends of the whiskers represent the minimum and maximum values of the data. Colony-forming units (CFU).

Among the microorganisms examined, bacteria dominated in the zoo air, constituting from 1.1 × 10^2^ to 3.0 × 10^3^ colony-forming units (CFU)/m^3^ in the employees’ area and from 2.2 × 10^3^ (E8) to 1.5 × 10^4^ CFU/m^3^ in the visitors’ area (Figure 3A, Table S2). The highest bacterial contamination (>10^4^ CFU/m^3^) was recorded at locations V4 and V5 (ground floor of elephants’ zone and macaques’ zone), which may be related to high dust levels in these places and the use of particles lifted from the ground as carried by microorganisms (Figure 3B, Table S2).

Fungi were less abundant in the tested samples—in the employees’ area, they ranged from 2.0 × 10^1^ to 1.4 × 10^3^ CFU/m^3^, and in the visitors’ area, from 1.4 × 10^1^ to 2.2 × 10^3^ CFU/m^3^ (Figure 3, Table S2). This group of microorganisms dominated in E3 and E4 (employees’ kitchen and employees’ cloakroom) and V7 (free flight zone—first floor) (Figure 3, Table S2). *Actinomycetes* were less abundant, constituting from 3.3 × 10^0^ to 1.9 × 10^3^ CFU/m^3^ (employees’ area) and 1.0 × 10^1^ to 5.2 × 10^2^ CFU/m^3^ (visitors’ area); mannitol-positive *Staphylococcus* spp.—from 3.3 × 10^0^ to 3.7 × 10^2^ CFU/m^3^ (employees’ area) and 1.3 × 10^1^ to 7.5 × 10^2^ CFU/m^3^ (visitors’ area); and hemolytic *Staphylococcus* spp.—from 1.0 × 10^1^ to 1.9 × 10^2^ CFU/m^3^ and 3.3 × 10^0^ to 3.6 × 10^2^ CFU/m^3^, respectively. The least abundant species in the zoo’s air was *Pseudomonas fluorescens*, detected in the visitors’ area in 8 of 12 samples at concentrations ranging from 3.3 × 10^0^ to 1.67 × 10^1^ CFU/m^3^, and in 4 of 12 locations (E4, E6, E7, and E10) in the employees’ area in the same concentration range (Figure 3A, Table S2). The presence of the Enterobacteriaceae family was also low. In the visitors’ area, the maximum was 2.5 × 10^1^ CFU/m^3^ (V5 and V7) (Figure 3B, Table S2). Enterobacteriaceae were recorded only in locations E5, E6, and E7, which may be concerning because these are primarily rooms intended for storing pet food and supplements, often equipped with refrigerators and freezers for storing foods that require low temperatures. The higher prevalence of Enterobacteriaceae in visitor areas is likely due to heavy human traffic, as these bacteria are common in the human gastrointestinal tract and are readily shed through fecal contamination, as well as through contact with contaminated surfaces such as barriers, railings, and windows frequently touched by visitors [20]. Furthermore, the presence of children, who are more prone to hand-to-mouth behavior, may contribute to the spread of these bacteria in public areas [36].

Statistical analysis of the number of microorganisms in the zoo’s air showed a significant difference (*p* < 0.05) depending on the sampling location, except for the number of *P. fluorescens* and Enterobacteriaceae counts in the employees’ area, for which no statistically significant differences were detected between the study locations. In the visitors’ area, greater statistical variation in the obtained results was detected, allowing us to identify locations with lower levels of fungal contamination, i.e., V3 and V5 compared to V7; *Actinomycetes*-V1 and V6 compared to V11; mannitol-positive *Staphylococcus* spp.-V1 compared to V6 and V7 and V12 compared to V7; and for hemolytic *Staphylococcus* spp.-V1 compared to V9. As in the employees’ area, there were no statistically significant differences in the number of *P. fluorescens*.

It should be emphasized that this analysis showed the lowest microbiological contamination in ambient air in the parking lot located within 15 m of the main entrance to the zoo. There is little research on microbiological air quality in zoos. Grzyb and Lenart-Boroń highlight this issue, pointing out that numerous studies have been published on bioaerosols in various environments related to animal husbandry (including farms, stables, barns, pigsties, and henhouses), but no comprehensive microbiological analyses have been conducted in zoos [30]. The most recent study by Plewa-Tutaj et al. focused solely on the analysis of fungal bioaerosols in the Wrocław Zoological Garden (Poland) [38]. Like the current study, the authors used the impact sampling method and examined 20 animal enclosures. They reported fungal concentrations in a wider range than ours, ranging from 5.0 × 10^1^ to 3.6 × 10^4^ CFU/m^3^. Previously, Grzyb and Lenart-Boroń assessed the number of fungi in the premises of the Krakow Zoo, obtaining results in a narrower range of 8.4 × 10^2^–1.3 × 10^3^ CFU/m^3^ [31].

Bacterial counts were also examined in the premises of the Zoos in Krakow and Chorzów [30,32]. They found a total count of mesophilic bacteria ranging from 8.5  ×  10^2^ to 1.1 ×  10^5^ CFU/m^3^ and a count of mannitol-positive staphylococci ranging from 2.2  ×  10^1^ to 5.7  ×  10^3^ CFU/m^3^, depending on the facility and the room (animals). In our study, we found lower counts of Mannitol-positive staphylococci. Similarly to the present study, the authors also noted statistically significant differences in the concentration of microorganisms between the zoo air and the control air [30,31]. It is worth emphasizing that our study provides the first data on the number of *Actinomycetes*, hemolytic *Staphylococcus* spp., *P. fluorescens*, and Enterobacteriaceae in zoo air. Many studies indicate correlations between the number of airborne microorganisms and environmental factors such as temperature and relative humidity, as well as seasonal variations in microbiological contamination [39,40]. Therefore, research aimed at monitoring the microbiological quality of air in zoos should be continued. This is important in the context of the specific nature of these places—the exposure of animal keepers on the one hand and visitors, often with small children, on the other. This is particularly important given children’s higher respiratory rate relative to body size, narrower airways, immature immune systems, and frequent hand-touching of the mouth [36,40,41,42]. Unlike occupational exposure, which is regulated and may be limited the use of PPE, visitor exposure (especially children) is not subject to established safety standards. This gap highlights the urgent need for evidence-based guidelines for microbiological air quality in public facilities that handle animals.

These analyses are also important given the current lack of standards establishing acceptable levels of microorganisms in such spaces. Directive 2000/54/EC concerns workers’ protection from microbiological hazard exposure at work, and the information provided is focused on general aspects, e.g., risk assessment (including classifications of biological agents, recommendation of workers protection, potential allergenic and toxigenic effects, and exposition to specific diseases related to work), risk reduction, and hygiene [21]. However, there is no specified information regarding the standardization of measurement methods for microbiological pollution monitoring, nor about their threshold limits.

The Expert Group on Biological Agents at the Polish Interdepartmental Commission for Maximum Admissible Concentrations and Intensities for Agents Harmful to Health in the Working Environment proposed threshold limit values for microorganisms in the air at workplaces polluted with organic dust and in outdoor (atmospheric) air [43]. Comparing the obtained results with the proposed limits, these values for bacterial and fungal concentrations were not exceeded. However, other proposed limit values can be found in the literature, such as those proposed by the Commission of the European Communities [44], which considers the air in the employees’ areas to be moderately polluted and the air in the visitors’ areas to be highly polluted with bacteria.

Considering the above, the safe ranges of airborne microbiological contamination for humans in animal environments have not been precisely established. Consequently, it is crucial to define the limits of microbiological agents in the air for people, including for workers closely exposed to animals in the workplace.

**Figure 4:**
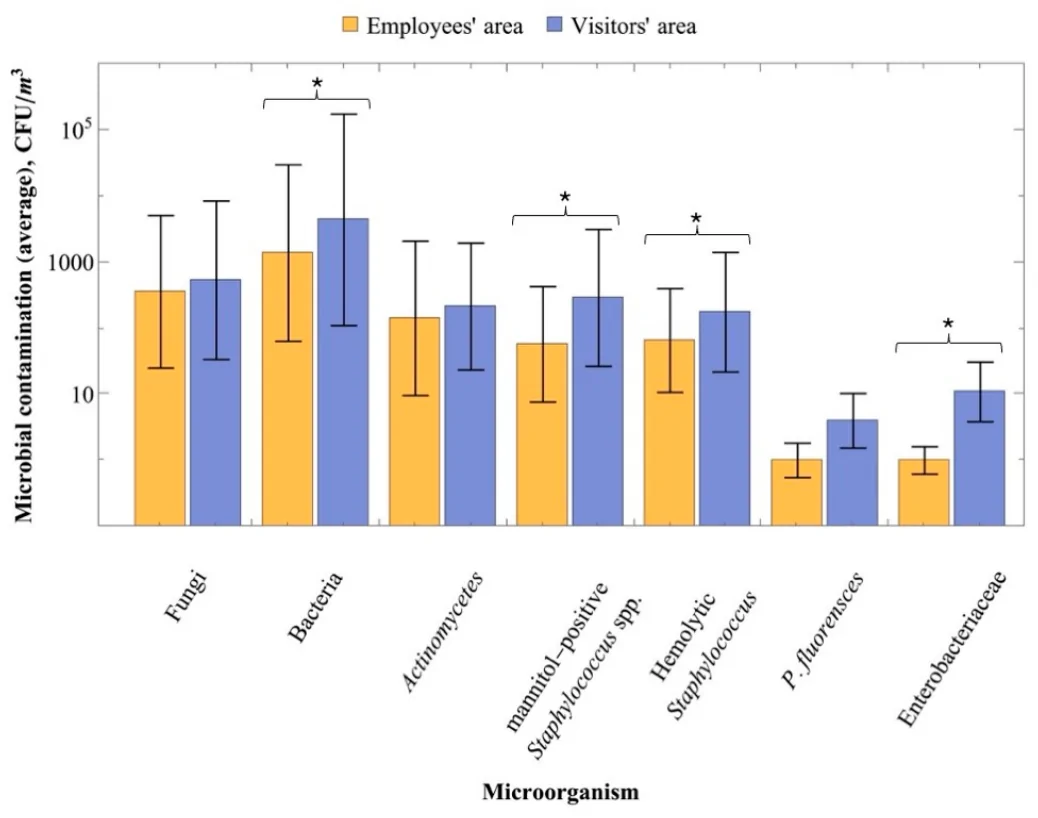
Comparison of airborne microbial contamination between employee and visitor areas. Bars represent the arithmetic means of location-level microbial concentrations (*n* = 12 sampling locations per area), and error bars indicate the standard deviation between sampling locations. Employee and visitor areas were compared separately for each microbial group using the Kruskal–Wallis test. An asterisk (*) indicates a statistically significant difference between employee and visitor areas (<0.05).

When location-level mean concentrations were compared using the Kruskal–Wallis test, with *p*-values adjusted for multiple comparisons using the Benjamini–Hochberg procedure, statistically significant differences between visitor and employee areas were found for total bacteria, mannitol-positive *Staphylococcus* spp., hemolytic *Staphylococcus* spp., and Enterobacteriaceae (*p* < 0.05; Figure 4). The mean total bacterial concentration was approximately 3.2-fold higher in visitor areas than in employee areas (4.5 × 10^3^ vs. 1.4 × 10^3^ CFU/m^3^, respectively). The mean concentrations of mannitol-positive *Staphylococcus* spp. and hemolytic *Staphylococcus* spp. were approximately 5.0-fold and 2.7-fold higher, respectively, in visitor areas. Enterobacteriaceae were also detected at a substantially higher mean concentration in visitor areas than in employee areas (11.3 vs. 0.8 CFU/m^3^). No statistically significant differences between the two areas were found for fungi, *Actinomycetes*, or *Pseudomonas fluorescens*. Overall, these findings show that visitor-accessible areas were characterized by higher concentrations of selected bacterial groups of potential health relevance, indicating a potentially greater opportunity for exposure to opportunistic microorganisms in these areas.

#### 2.4.2. Surface Microbial Contamination

**Figure 5:**
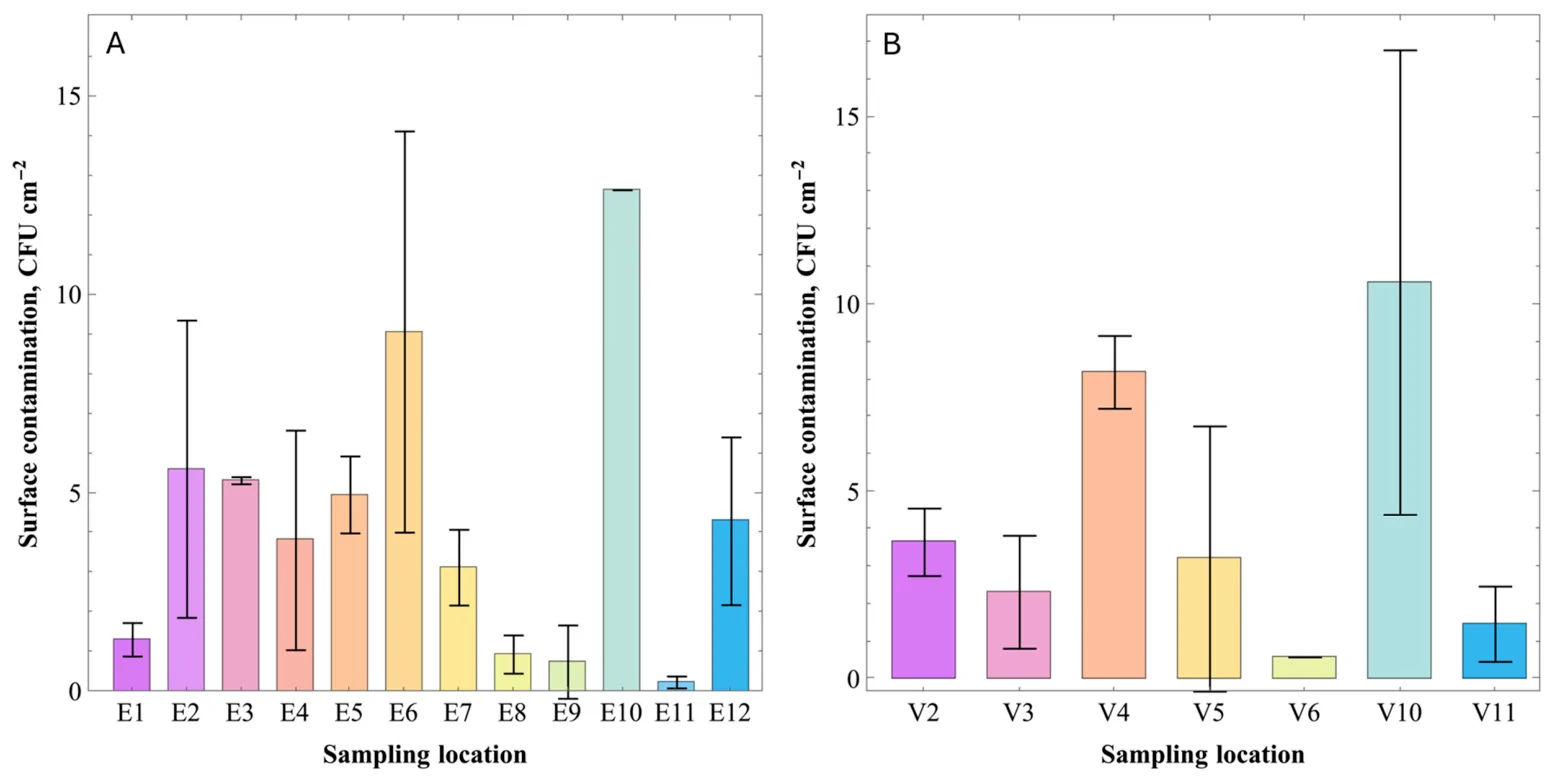
Surface contamination; (**A**) employees’ area and (**B**) visitors’ area.

Microbiological surface contamination (total number of bacteria and fungi) in the studied areas ranged from 0.1 × 10^0^ CFU/cm^2^ (glass doors in the hallway, office workers and animal workers, and aquarium window in the scuba divers’ zone) to 1.3 × 10^1^ CFU/cm^2^ (space for food storage on employees’ desks) in employees’ areas (Figure 5). In areas accessible to visitors, the average surface contamination ranged from 0.58 × 10^0^ (barrier in the free flight area) to 1.6 × 10^1^ CFU/cm^2^ (elephant bathing area). The average number of microorganisms in the employees’ and visitors’ areas was the same (4.3 CFU/cm^2^) (Figure 5).

To our knowledge, we present the first study of microbiological surface contamination in a zoo. Aksoy et al. studied microbiological contamination in a veterinary hospital using contact plates but focused exclusively on enumerating *Staphylococcus* spp. [45]. Similar studies of pathogens in such facilities were conducted by Singaravelu et al. [46] and Spratt et al. [47]. Other authors focused on determining the optimal method for monitoring and disinfecting surfaces in agricultural and veterinary environments, e.g., using boot swab sampling [48,49]. Alsing-Johansson et al. examined 276 samples in dog cages using a sampling sponge and Petrifilm™ AC plate [49]. They observed microbial contamination in wounds ranging from 0 to 1.3 × 10^4^ CFU/cm^2^ on floors and from 0.6 × 10^0^ to 3.2 × 10^1^ CFU/cm^2^ on walls, which appears similar to our results. As with the number of microorganisms in the air, there are no legally permissible limits for surface contamination. The data obtained in this study can also be compared to analyses conducted in other public facilities, e.g., museums, archives, and libraries, where the number of microorganisms was recorded at levels of 1.4 × 10^0^–1.7 × 10^2^ CFU/cm^2^ and 8.4 × 10^0^–8.8 × 10^0^ CFU/cm^2^ [50], or to the surfaces of waiting, consulting, and sterilization rooms of dental and general practitioner offices, where the number of microorganisms ranged from 0 to 1.3 × 10^3^ CFU/cm^2^. Considering the above literature values, it can be concluded that the microbiological contamination of the tested surfaces in the zoo was at a non-specific level and did not indicate an increased level of microbiological threats in this environment.

#### 2.4.3. Protective Goggles Microbial Contamination

**Figure 6:**
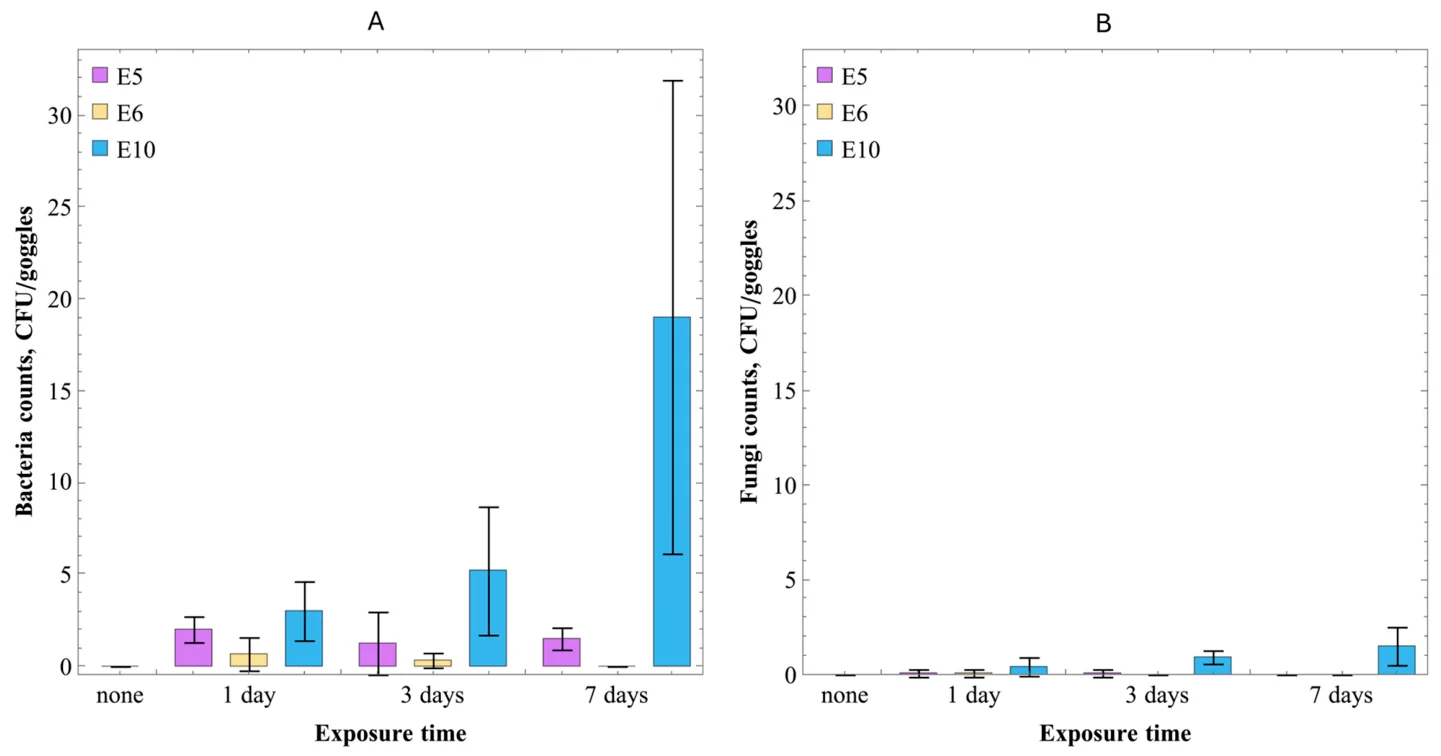
Protective goggle microbial contamination; (**A**) bacteria and (**B**) fungi.

In our study, we found contamination of goggles in the zoo’s work environment at 0–1.9 × 10^2^ CFU/goggles (bacteria) and 0–1.5 × 10^0^ CFU/goggles (fungi) (Figure 6). The highest microbial counts were recorded after 7 days of exposure in the elephants’ headquarters. However, because each workplace–exposure time combination was represented by a single independent pair of goggles, the results are descriptive and do not establish a statistically significant time-dependent trend. To date, contamination of protective goggles has only been studied in the context of their safe use and disinfection by healthcare workers. The authors of these studies focused primarily on the presence of pathogenic microorganisms and their percentage in the tested samples [51]. Fritz et al. examined spectacles used at universities and nursing homes, finding a median contamination of 1.4 × 10^3^–20.8 × 10^3^ CFU/cm^2^ [52]. Our research is new in terms of assessing the contamination of protective equipment such as goggles in work environments involving the presence of animals.

#### 2.4.4. Settled Dust Microbial Contamination

**Figure 7:**
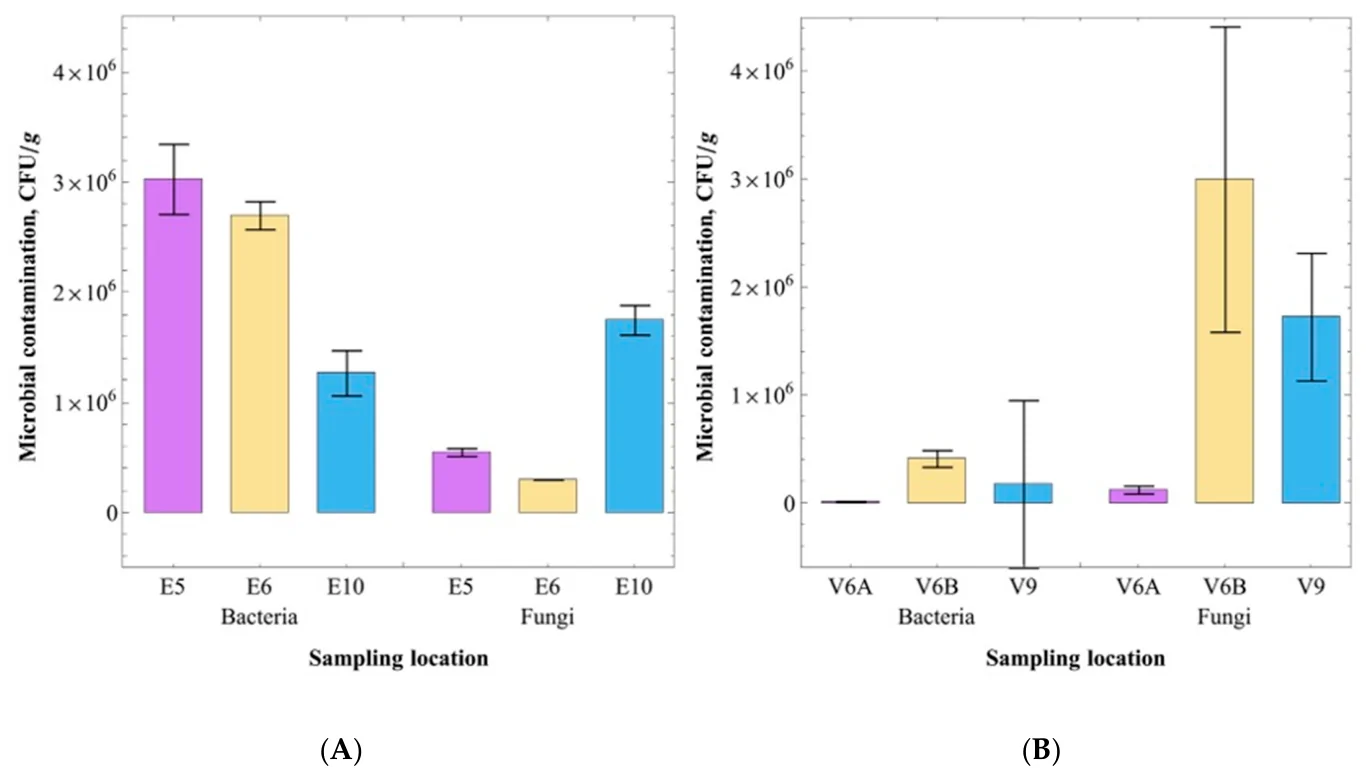
Settled dust microbial contamination; (**A**) employees’ area and (**B**) visitors’ area.

Settled dust was analyzed as an environmental matrix complementary to active air sampling and was not intended to represent the inhalable airborne fraction. Microbiological contamination of settled dust collected at the zoo ranged from 2.7 × 10^6^ to 1.1 × 10^7^ CFU/g (bacteria) and 3.0 × 10^5^ to 1.8 × 10^6^ CFU/g (fungi) in employees’ areas (Figure 7A). In samples from visitors’ areas (Figure 7B), the contamination of dust for bacteria and fungi ranged from 1.6 × 10^4^ to 4.2 × 10^5^ CFU/g and 1.2 × 10^5^ to 3.0 × 10^6^ CFU/g, respectively (Figure 7B). Currently, no other data on microbiological contamination of settled dust from the zoo premises is available. However, Gutarowska et al. conducted extensive research in this area at a cement plant, composting plant, poultry farm, and cultivated area [53]. They found the number of bacteria in the dust samples ranged from 3.8 × 10^2^ to 1.6 × 10^8^ CFU/g and the number of fungi ranged from 1.5 × 10^2^ to 6.5 × 10^6^ CFU/g, depending on the sampling location, with the lowest microorganism counts observed at a cement plant. Skóra et al. described microorganism concentrations in the settled dust in poultry farms (3.2 × 10^9^ CFU/g for bacteria and 1.2 × 10^6^ CFU/g for fungi) [54]. This suggests that the microorganism levels observed in settled dust in this study do not differ significantly from those observed in farm buildings.

### 2.5. Microbial Community Composition and Diversity

At the phylum level, the bacterial community was dominated by Bacillota (55.1% in the visitors’ area and 53.3% in the employees’ area), followed by Pseudomonadota (the second most abundant phylum in the visitors’ area) (18.7% vs. 21.6%) and Actinomycetota (the second most abundant phylum in the employees’ area) (19.7% vs. 16.4%). Bacillota and Actinomycetota were more abundant in the visitors’ area, while Pseudomonadota and Bacteroidota (2% vs. 3.5%) were elevated in the employees’ area. Several minor phyla (e.g., Bacillota C, Myxococcota, and Bacillota I) were detected only in the employees’ samples, whereas Gemmatimonadota and Chloroflexota were exclusive to the visitors’ area.

**Figure 8:**
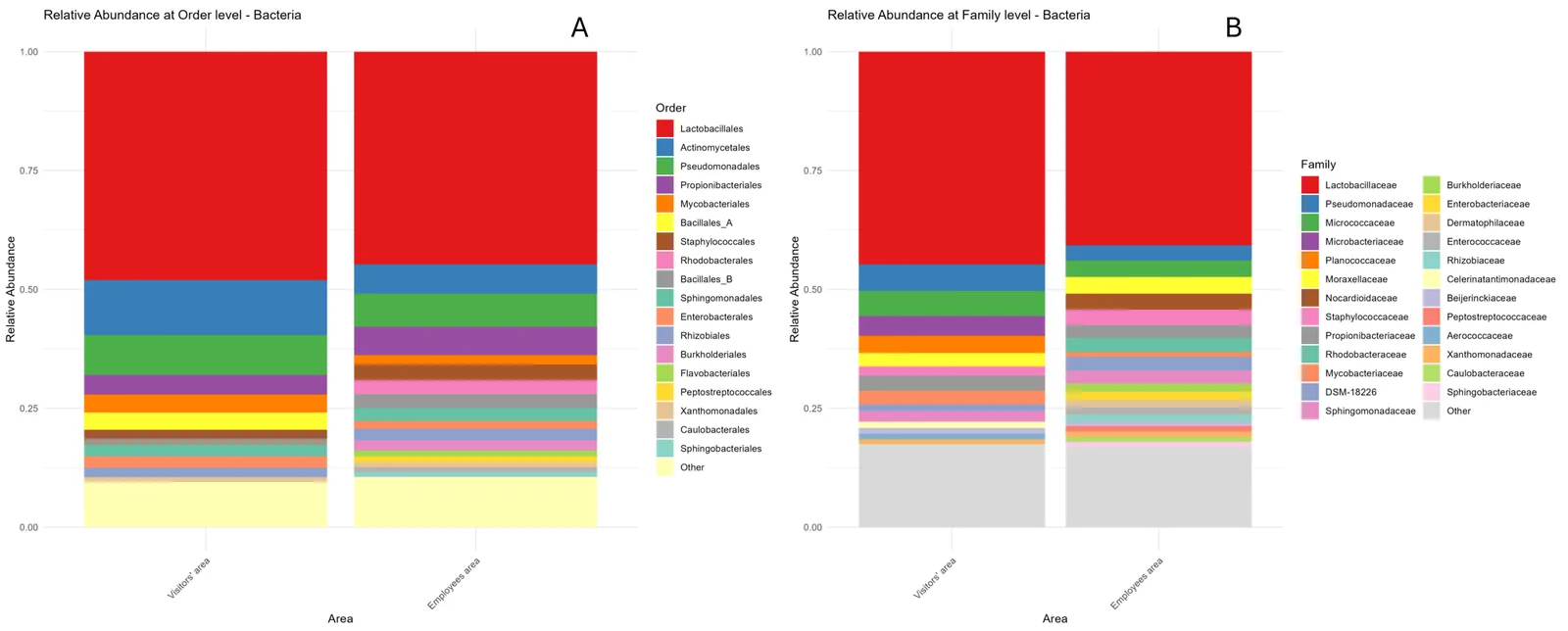
The relative abundance of bacterial taxa; (**A**) by order and (**B**) family in air samples from visitors’ and employees’ areas.

At the class level, the leading class in both areas was Bacilli (55.1% vs. 53.3%), consistent with the dominance of Bacillota. *Actinomycetes* (19.5% vs. 14.5%) and Gammaproteobacteria (12.9% vs. 11.8%) were substantial components, with *Actinomycetes* noticeably higher in the visitors’ area. Alphaproteobacteria and Bacteroidia increased markedly in the employees’ area (5.9% vs. 9.8% and 2% vs. 3.5%, respectively). At the order level (Figure 8A), two orders dominated both environments: Lactobacillales (48% in the visitors’ area and 44.8% in the employees’ area) and Actinomycetales (11.5% vs. 6.2%). Pseudomonadales, Mycobacteriales, and Enterobacterales were present in both areas but were lower in the employees’ area. Orders enriched in the employees’ area included Propionibacteriales, Sphingomonadales, Rhizobiales, Rhodobacterales, Bacillales B, Staphylococcales, and Burkholderiales. Only a few orders were unique to a single environment, indicating high overlap at this level. At the family level (Figure 8B), the dominant family was Lactobacillaceae (44.7% in the visitors’ area and 40.7% in the employees’ area). Pseudomonadaceae, Micrococcaceae, Microbacteriaceae, Planococcaceae, and Mycobacteriaceae were consistently higher in the visitors’ area. Propionibacteriaceae were slightly higher in the visitors’ area. In contrast, employees’ samples showed elevated clusters of Staphylococcaceae, Rhodobacteraceae, Nocardioidaceae, and Peptostreptococcaceae, all of which were low in the visitors’ area.

**Figure 9:**
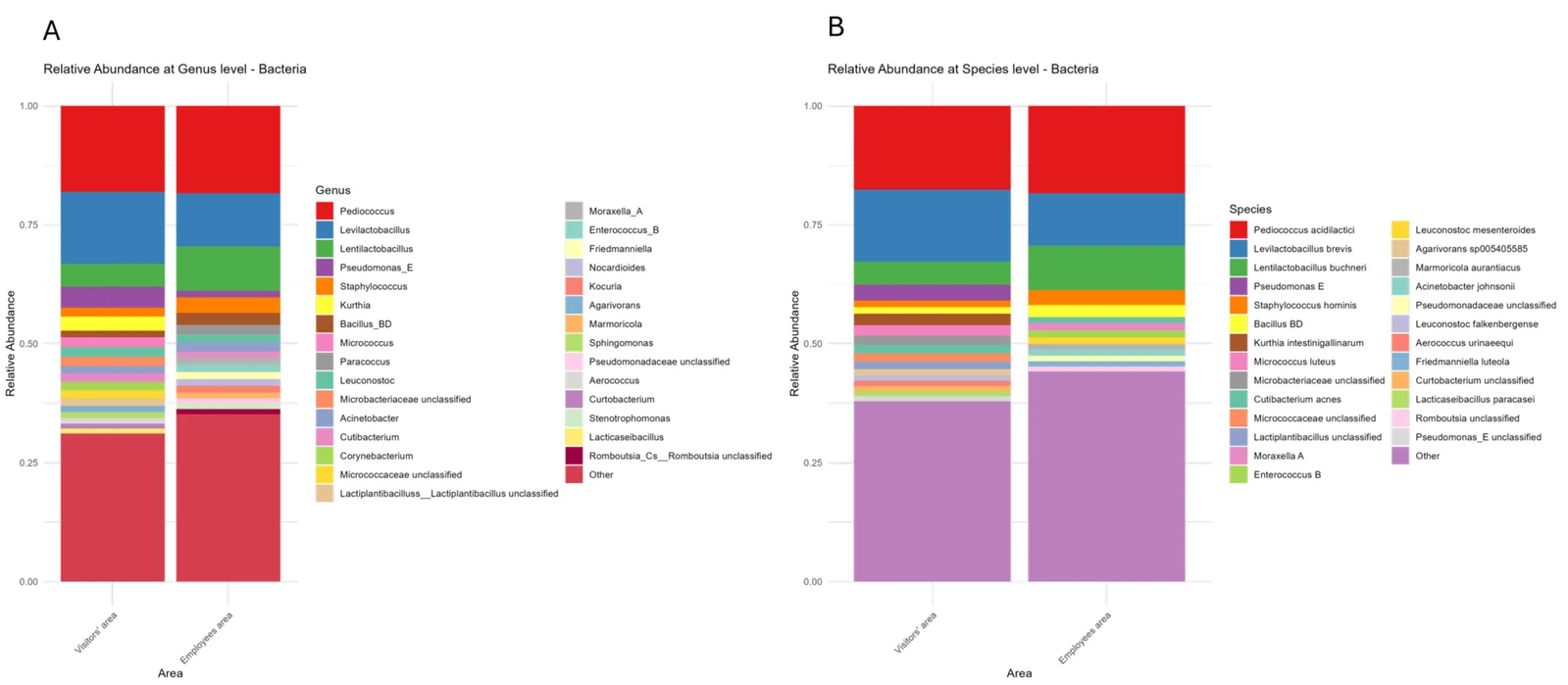
The relative abundance of bacterial taxa; (**A**) by genus and (**B**) species in air samples from visitors’ and employees’ areas.

At the genus level (Figure 9A), *Pediococcus* was the single largest cluster in both environments (18.1% in the visitors’ area and 18.4% in the employees’ area). *Levilactobacillus* was the second most abundant genus, yet its relative abundance varied from 15.1% in the visitors’ area to 11.2% in the employees’ area. Conversely, *Lentilactobacillus* nearly doubled in the employees’ area (from 4.8% to 9.4%), indicating a shift within Lactobacillaceae. *Staphylococcus* was also higher (1.9% vs. 3.3%). Genera such as *Pseudomonas E*, *Leuconostoc*, *Micrococcus*, and *Cutibacterium* were present in both areas but generally higher in the visitors’ area. The employees’ samples contained markedly enriched genera, including *Moraxella A*, *Paracoccus* and *Nocardioides*. At the finest taxonomic resolution (Figure 9B), the most abundant cluster in both areas was *Pediococcus acidilactici* (17.6% in the visitors’ area and 18.4% in the employees’ area). *Levilactobacillus brevis* (15.1% and 11%) and *Lentilactobacillus buchneri* (4.8% and 9.4%) were next in rank. *Staphylococcus hominis*, *Bacillus* BD, and *Moraxella* A were notably enriched in the employees’ samples, while *Pseudomonas* E and *Kurthia intestinigallinarum* were common only in the visitors’ area. Several species appeared exclusively in one environment, illustrating habitat-specific signatures.

In fungi at the phylum level, *Ascomycota* accounted for 83.7% of fungi in the visitors’ area and an even higher 90.4% in the employees’ area, indicating that these fungi overwhelmingly dominate both environments. *Basidiomycota* contributed 16% of clusters in the visitors’ area but only 9.6% of clusters in the employees’ area. At the class level, *Saccharomycetes* formed the largest cluster group in both samples (48.3% in the visitors’ area and 53.8% in the employees’ area). *Eurotiomycetes* (21% vs. 12.4%) and *Dothideomycetes* (10.4% vs. 8%) were abundant in both areas, yet more abundant in the visitors’ area and decreased in the employees’ area. Conversely, *Sordariomycetes* increased markedly from 2.8% in the visitors’ area to 14.4% in the employees’ area, becoming the second most abundant class in the employees’ area.

**Figure 10:**
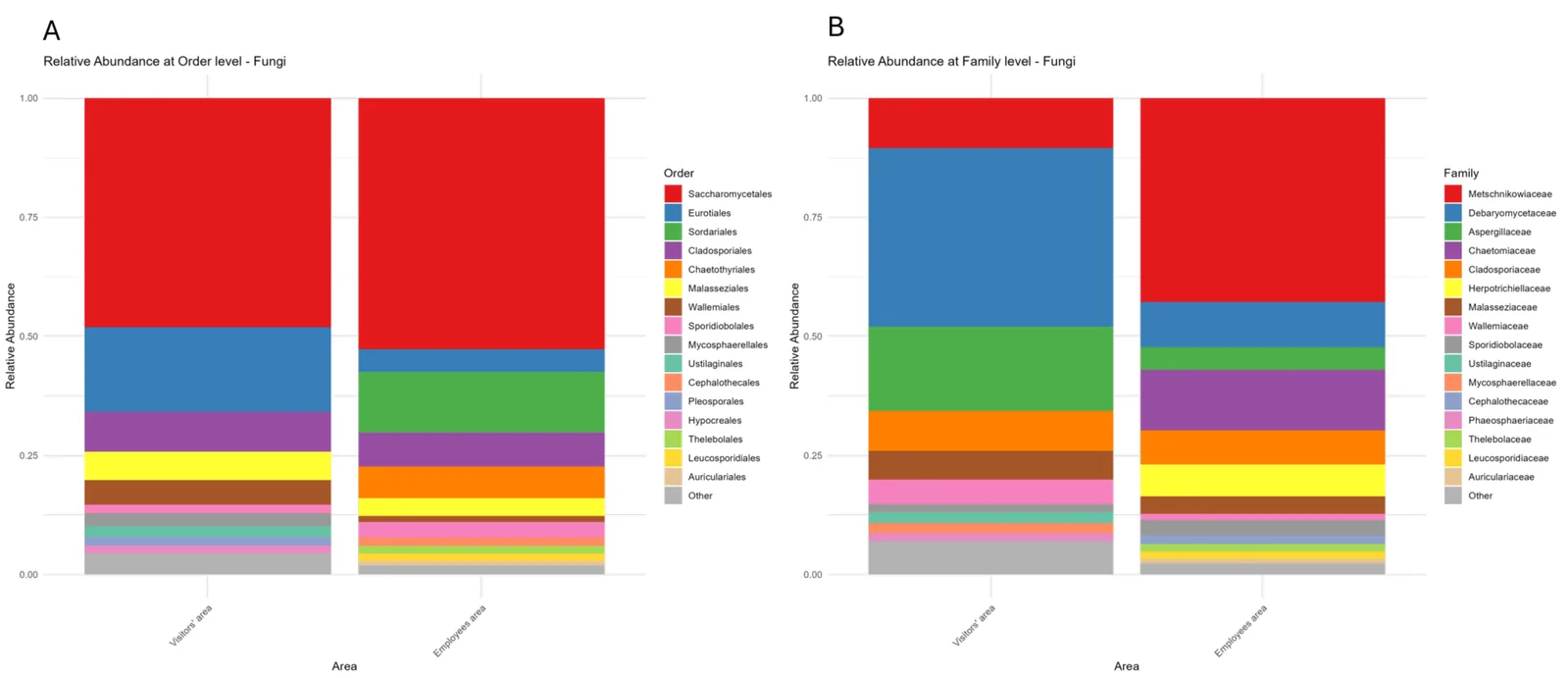
The relative abundance of fungal taxa; (**A**) by order and (**B**) family in air samples from visitors’ and employees’ areas.

The order-level distribution (see order-level stacked bar chart below) shows shifts between the two environments (Figure 10A). Saccharomycetales clusters were dominant in both areas (48.3% in the visitors’ area and 53.8% in the employees’ area). In the visitors’ area, Eurotiales (18.9%) and Cladosporiales (6.1%) comprised a large proportion of clusters, while Wallemiales (5.4%) and Malasseziales (6.0%) were also notable. In contrast, the employees’ area displayed substantial increases in Sordariales (1% vs. 11.7%), Chaetothyriales (2% vs. 7.4%), and Sporidiobolales (1.8% vs. 3.2%). In the employees’ area, Cladosporiales comprised 7.1% of all fungi (more than in the visitors’ area). Several orders were unique to the visitors’ area (e.g., Mycosphaerellales, Ustilaginales, Tremellales, and Pezizales) or to the employees’ area (e.g., Cephalothecales, Leucosporidiales, and Auriculariales).

Family-level patterns highlight contrasting dominant families between the two areas. In the visitors’ air samples, Debaryomycetaceae (37.4%) and Aspergillaceae (18.3%) were the major contributors, accompanied by Cladosporiaceae (6.1%) and Malasseziaceae (6.0%) (Figure 10B). The employees’ air samples, however, were dominated by Metschnikowiaceae (42.7%). The employees’ samples also contained high proportions of Chaetomiaceae (11.7%), Debaryomycetaceae (9.5%), and Herpotrichiellaceae (7.4%), with the Chaetomiaceae and Herpotrichiellaceae families being minor in the visitors’ area. Families such as Mycosphaerellaceae, Ustilaginaceae, Phaeosphaeriaceae, and Pezizaceae were present only in the visitors’ area, whereas Cephalothecaceae, Leucosporidiaceae, and Auriculariaceae were exclusive to the employees’ area.

**Figure 11:**
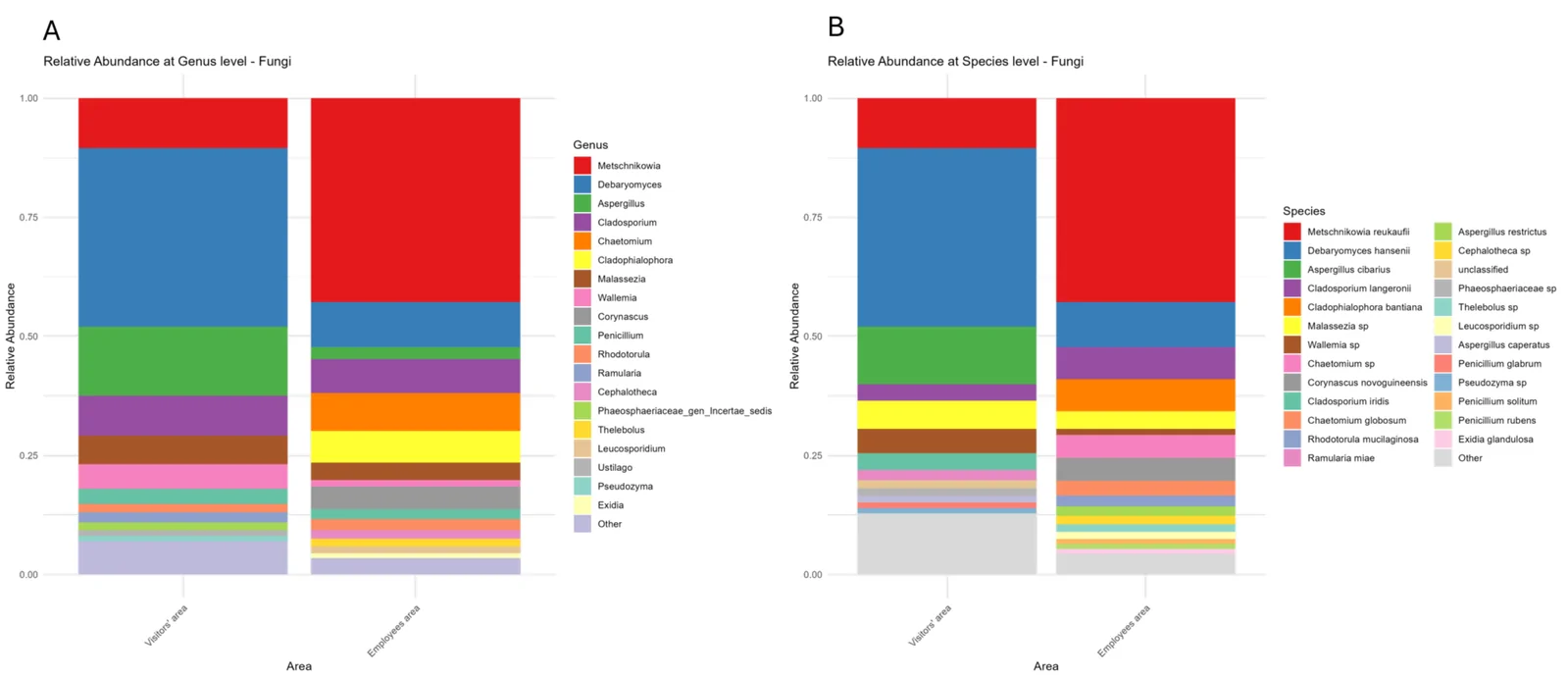
The relative abundance of fungal taxa; by genus (**A**) and species (**B**) in air samples from visitors’ and employees’ areas.

The genus-level composition reveals a striking shift (Figure 11A). Among air samples from the visitors’ area, *Debaryomyces* (37.4%) was the most abundant genus, followed by *Aspergillus* (15.6%), *Metschnikowia* (10.5%), *Cladosporium* (6.1%), and *Malassezia* (6.0%). In the employees’ air samples, *Metschnikowia* increased dramatically to 42.7% and became the dominant genus, followed by *Debaryomyces* (9.5%), *Cladophialophora* (7.4%), and *Cladosporium* (7.1%). Besides *Metschnikowia* and *Cladophialophora*, *Chaetomium* and *Corynascus* also rose sharply in the employees’ area, while (aside from *Debaryomyces*) *Aspergillus* and *Wallemia* declined. Unique genera in the visitors’ area included, among others, *Ramularia*, *Pseudozyma*, *Papiliotrema*, *Ustilago* and *Peziza*, whereas the employees’ area featured *Corynascus*, *Sporobolomyces*, *Cephalotheca*, *Leucosporidium*, *Exidia*, *Fusarium*, and *Scopulariopsis*—genera absent in the visitors’ area.

At the species level (Figure 11B), a single yeast dominated each environment but differed between them. *Debaryomyces hansenii* was the top species in the visitors’ area (37.4%), followed by *Aspergillus cibarius* (12.1%), *Metschnikowia reukaufii* (10.5%), *Malassezia* sp. (5.9%), *Wallemia* sp. (5.4%), and *Cladosporium iridis* (4.0%). *Metschnikowia reukaufii* dominated the employees’ area (42.7%), followed by *Debaryomyces hansenii* (9.5%), *Cladophialophora bantiana* (7.4%), *Cladosporium langeronii* (6.8%), *Corynascus novoguineensis* (4.7%), and *Chaetomium* sp. (4.0%). All other species fell below 4%. Notably, *Cladosporium iridis* was absent in the employees’ area, whereas *Corynascus novoguineensis* was absent in the visitors’ area. The species showing the greatest enrichment in the visitors’ area were *Debaryomyces hansenii* and *Aspergillus cibarius* (12.1% vs. 0.2%), whereas the species most enriched in the employees’ area were *Metschnikowia reukaufii*, *Cladophialophora bantiana* (1.4% vs. 7.4%), and *Cladosporium langeronii* (1.7% vs. 6.8%).

**Figure 12:**
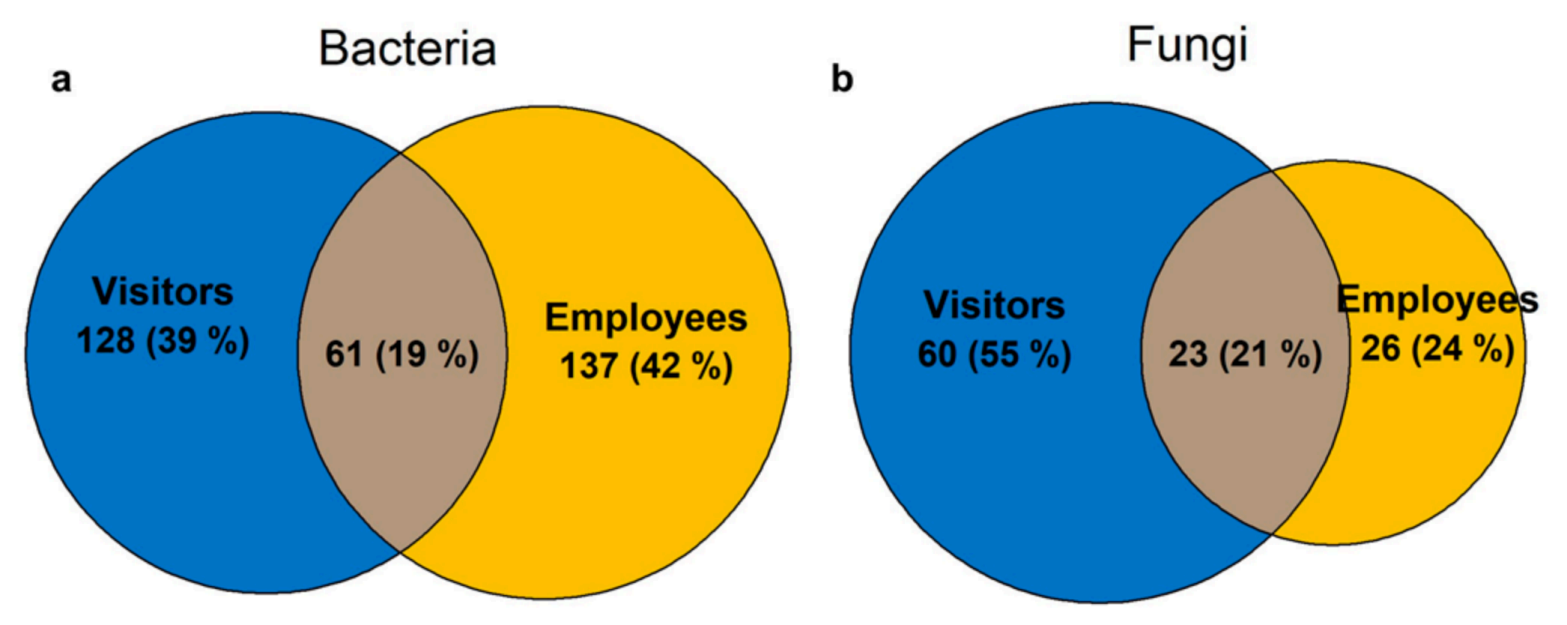
Venn diagrams comparing the clusters detected in the visitors’ and employees’ air samples: (**a**) shows the number and percentage of unique and shared bacterial clusters; (**b**) shows the corresponding comparison for fungal clusters.

Figure 12a compares the bacterial clusters detected in visitors’ and employees’ air samples. It shows that 128 clusters (39%) were unique to the visitors’ area, 137 clusters (42%) were unique to the employees’ area, and 61 clusters (19%) were shared by both, indicating a modest core microbiome and many site-specific clusters. Figure 12b depicts the fungal clusters; 60 fungal clusters (55%) were exclusive to the visitors’ area, 26 clusters (24%) were exclusive to the employees’ area, and 23 clusters (21%) were shared. This indicates that the visitors’ area harbored substantially more unique fungal diversity compared to the employees’ area, with the employees’ area and shared clusters contributing roughly equally to the remaining fungal community.

To date, studies of microbial diversity in zoos have focused on assessing the fecal microbiota of animals housed there in relation to their wild counterparts, diet, or environmental conditions, concentrating on individual animal groups and the factors shaping their microbiota [55,56,57,58].

To the best of our knowledge, the study we describe is the first to comprehensively depict air biodiversity in zoos and to utilize nanopore sequencing for this purpose. In the zoo environment, we identified lactic acid bacteria, including *Pediococcus* (dominantly *P. acidilactici*), *Levilactobacillus* (*L. brevis*), *Lentilactobacillus* (*L. buchneri*), and *Leuconostoc*. Their presence is unsurprising, as they are part of the natural microbiota of animals, humans, and fermented plant materials, often possessing probiotic properties [59].

In both areas (with a predominance in the workers’ area), we also identified *Micrococcus*, *Staphylococcus* (mainly *S. hominis*), *Pseudomonas E*, *Cutibacterium*, and *Kurthia intestinigallinarum*. *Micrococcus* and *Staphylococcus hominis* cocci are representative of the microbiota of the skin, of humans and animals (preferentially axillae, arms, legs, and pubic and inguinal regions), including saprophytes and pathogenic strains [60,61]. *Pseudomonas* species typically colonize moist surfaces and have been documented in indoor air. They are particularly important in healthcare settings because they include opportunistic pathogens such as *P. aeruginosa* [62]. *Cutibacterium* (formerly *Propionibacterium*) is a cutaneous corynebacterium, among which strains of opportunistic pathogens have been identified that cause superficial or deep/invasive infections [63].

*Kurthia* has previously been isolated from soil, meat, and feces, while *K. intestinigallinarum* has been isolated from the oral cavity of a deer [64]. These bacteria are suspected of causing meat spoilage and have pathogenic potential but have not yet been thoroughly studied [64].

*Bacillus* BD and *Moraxella* were more common in the employees’ area, with *Bacillus* spp. being cosmopolitan, mostly saprophytic bacteria, often isolated from air [65].

*Moraxella* spp., on the other hand, is frequently isolated from clinical samples (humans and animals). However, most of these bacteria are harmless and constitute a significant portion of the psychrotrophic aerobic flora of many fresh and spoiled food products, such as meat, fish, cheese, milk, and vegetables. Among the pathogens of this type of microbe, *M. catarrhalis* is known, which can cause otitis media, upper respiratory tract infections in children and the elderly, and lower respiratory tract infections in adults [66].

In the zoo environment, we demonstrated the dominance of the following fungi: *Debaryomyces hansenii*, *Aspergillus cibarius*, *Metschnikowia reukaufii*, *Malassezia*, *Wallemia*, and *Cladosporium iridis*, *C. bantiana*, *C. langeronii*, *Corynascus novoguineensis*, and *Chaetomium*.

*Debaryomyces hansenii* is characteristic of various natural sources, including fruit, air, water, and soil, but most commonly from processed foods. It has also been isolated from humans and animals [67]. All this makes its presence in the zoo’s air not surprising.

*Metschnikowia reukaufii* is a nectar-borne yeast and occurs in the air of flower-rich areas [68], which was the case in the study. *Aspergillus cibarius* was isolated from meju (a brick of dried fermented soybeans), black beans, bread, and salami [69]. Its presence in the zoo’s facilities most likely results from the use of soy or fermented feed in animal feeding.

*Malassezia* spp. are lipolytic commensal fungi commonly found on human and animal skin (mammals and birds), which also play a pathogenic role in various skin diseases [70,71].

*Wallemia* belongs to extremophiles (xerophiles and halophiles)–it occurs in air, soil, dried food (which causes spoilage), and salt [72]. Its airborne source is most likely saltwater, which is the habitat of many animals in the oceanarium at the studied facility.

Fungi of the genus *Cladosporium* are part of the microbiota of the natural environment. Ghiaie et al. studied air samples in Iran, demonstrating that *Cladosporium iridis* was present in over 44% of cases (44.3%) [73]. Similarly, *C. langeronii* is a xerogenic fungus common in the natural environment (including dead plant matter, soil, and air) [74]. However, it is worth noting the species *C. bantiana*, which has been described in the literature as pathogenic to humans and animals. Most *C. bantiana* infections affect the central nervous system, causing mycosis of the brain; it can also cause cutaneous or subcutaneous infections, pulmonary infections, sinusitis, arthritis, and osteomyelitis [75]. Cases of *C. bantiana* infections have been described in animals, including horses, cats, and dogs [76]. *Corynascus novoguineensis* and *Chaetomium* belong to the Chaetomiaceae family and act as saprophytes, participating in the decomposition of organic matter. They are most often isolated from cellulosic substrates, soil, and also air and dust in the case of *Chaetomium* [77,78].

Previously, only Plewa-Tutaj et al. attempted to isolate and identify fungi from the Wrocław Zoological Garden [38]. Using both morphological and molecular methods based on sequencing regions of the internal transcribed spacer (ITS), *β-tubulin*, and *calmodulin*, they identified 112 fungal strains belonging to 50 species and 10 genera. They indicated that *Penicillium* was the dominant genus (58.9% of isolated strains), followed by Aspergillus (25.9%), *Cladosporium* (3.6%), *Talaromyces* (3.6%), Mucor (1.8%), and *Schizophyllum* (1.8%). Using the same methods, 18 *Aspergillus* isolates were identified, indicating their threat to human and animal health [79]. Our research therefore provides significantly expanded knowledge regarding the diversity of microorganisms in the zoo facilities.

### 2.6. Cytotoxicity of Settled Dust

**Figure 13:**
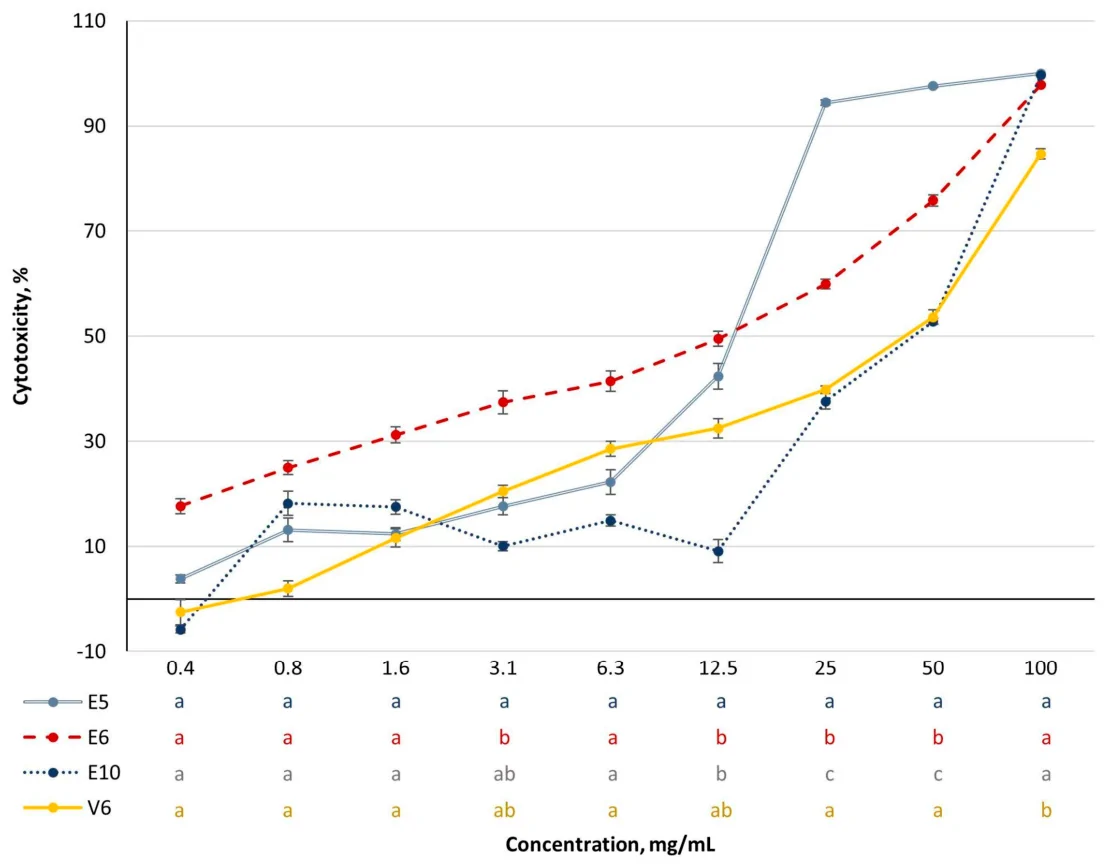
Cytotoxicity of settled dust as a function of sampling location: letters a–c represent statistical groupings based on one-way analysis of variance (ANOVA) followed by Tukey’s post hoc test. Within the same concentration, identical letters indicate no statistically significant difference, while different letters denote a significant difference (*p* < 0.05).

Treatment of A-549 cells with settled dust samples (0.4–100 mg/mL) resulted in a dose-dependent decrease in cell viability as measured by the 3-(4,5-dimethylthiazol-2-yl)-2,5-diphenyltetrazolium bromide (MTT) assay after 24 h exposure (Figure 13). At the highest settled dust concentration (100 mg/mL), cytotoxicity ranged from 84.7 to 100% after 24 h of treatment, for free flight area (visitors’ area) and workshop (employees’ area), respectively. The half-maximal inhibitory concentration (IC_50_) values, which indicate the concentration required to inhibit biological activity by 50%, were derived from dose–response curves after 24 h of exposure. Settled dust samples collected from the food storage (IC_50_ = 13.1 mg/mL) and workshop (IC_50_ = 14.3 mg/mL) exhibited the highest cytotoxicity toward A549 cells. In contrast, samples from the free flight zone (IC_50_ = 43.4 mg/mL) and the elephant headquarters (IC_50_ = 53.5 mg/mL) showed considerably lower cytotoxicity.

The four location-level IC_50_ values indicated considerable variation in the cytotoxicity of settled dust. The observed differences may reflect variation in the chemical and biological composition of the dust; however, the relative contributions of these components could not be determined in the present study. A formal correlation analysis between IC_50_ values and culturable microbial counts was not performed because only four location-level IC_50_ estimates were available, which was insufficient for a robust statistical assessment. Moreover, amplicon-sequencing data were obtained from bulk air samples rather than from the corresponding settled-dust samples; therefore, direct associations between cytotoxicity and dominant microbial taxa could not be evaluated. In the study of Plewa-Tutaj et al., fungal cytotoxicity was assessed on swine kidney cells using the MTT test, where strains with the highest cytotoxicity belonged to the *Aspergillus* section Fumigati isolated from Zoological Garden (Wrocław, Poland) [79]. When comparing the cytotoxicity with various animals’ habitats, e.g., poultry farms [53], dust cytotoxicity was calculated as 62% after 72 h of exposition.

### 2.7. Relationships Between Physicochemical and Microbiological Parameters

**Figure 14:**
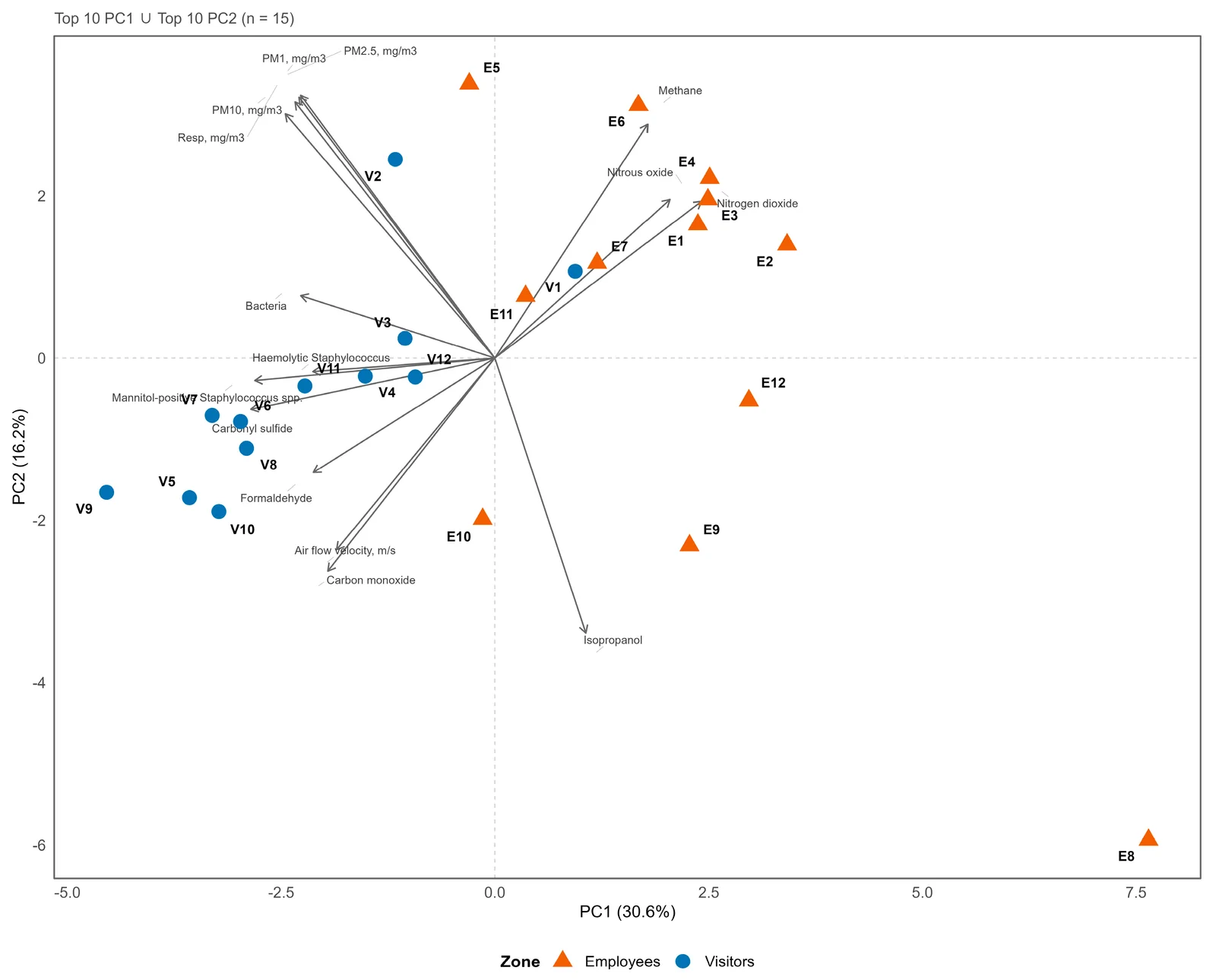
Principal component analysis (PCA) biplot of physicochemical and microbiological variables across zoo sampling sites. Arrows represent variable loadings on PC1 and PC2; arrow direction and length indicate the strength and direction of each variable’s contribution. Triangles denote employee areas; circles denote visitor areas. Data were log10-transformed (for concentration-based variables) and autoscaled prior to analysis. Variables shown represent the union of the 10 highest contributors to PC1 and PC2 (*n* = 15).

Principal component analysis (PCA) was performed to explore multivariate relationships among physicochemical and microbiological variables and differences in environmental profiles among zoo sampling sites (Figure 14). The first three principal components explained 61.4% of the total variance (PC1: 30.6%, PC2: 16.2%, and PC3: 14.6%). Principal component analysis (PCA) revealed a clear separation of sampling sites along PC1 according to zone type: employee areas were positioned predominantly at positive PC1 values, while visitor areas clustered at negative values. This separation was formally confirmed by permutational multivariate analysis of variance (PERMANOVA) (pseudo-F = 6.32, R^2^ = 0.223, *p* = 0.0001, 9999 permutations). The assumption of homogeneous multivariate dispersion was met (betadisper: F = 1.79, *p* = 0.194), confirming that the PERMANOVA result reflects genuine differences rather than differences in within-group variability.

The variables contributing most strongly to PC1 included carbonyl sulfide (contribution: 8.2%), mannitol-positive *Staphylococcus* spp. (8.0%), PM_10_ (6.1%), nitrogen oxides (5.9%), respirable dust (5.5%), and total bacteria (5.2%). Notably, microbiological and chemical variables co-varied along PC1, suggesting a common multivariate environmental gradient: carbonyl sulfide, PM fractions, mannitol-positive *Staphylococcus* spp., hemolytic *Staphylococcus* spp., total bacteria, and formaldehyde loaded together in one direction, while nitrogen dioxide, acetone, nitrous oxide, and methane loaded in the opposite direction. PC2 (16.2%) was primarily driven by PM fractions (PM_1_: 10.6%, PM_2.5_: 10.5%, RESP: 10.1%, and PM_10_: 9.2%) and isopropanol (11.6%), reflecting a gradient from high-dust environments to lower-dust zones. PC3 (14.6%) captured variations in air organic pollutants, principally methylmercaptan (19.5%), acrolein (18.0%), ammonia (14.0%), acetone (9.1%), and benzene (8.9%).

The administration office (E8) emerged as an outlier in PCA space (PC1 = 7.65, PC2 = −5.94), separated from all other samples. This position suggests its distinct environmental profile.

To quantify specific associations between physicochemical and microbiological variables, Spearman’s rank correlation coefficients were calculated for all 140 pairwise combinations of 20 physicochemical and 7 microbiological variables, with *p*-values corrected for multiple testing using the Benjamini–Hochberg procedure. Ten correlations remained significant after correction.

The strongest association was observed between carbonyl sulfide and mannitol-positive *Staphylococcus* spp. (ρ = 0.744, *p* adj. = 0.004), followed by a negative correlation between methane and Enterobacteriaceae and a positive correlation between cyclohexane and total bacteria (ρ = 0.672, *p* adj. = 0.015). Carbonyl sulfide additionally correlated with hemolytic *Staphylococcus* spp. (ρ = 0.633, *p* adj. = 0.032) and Enterobacteriaceae (ρ = 0.599, *p* adj. = 0.036).

Methane was negatively correlated with both Enterobacteriaceae (ρ = −0.696, *p* adj. = 0.011) and mannitol-positive *Staphylococcus* spp. (ρ = −0.603, *p* adj. = 0.036), indicating a negative association between methane concentrations and the abundance of these bacterial groups. Further significant associations included benzene and hemolytic *Staphylococcus* spp. (ρ = −0.598, *p* adj. = 0.036), nitrous oxide and Enterobacteriaceae (ρ = −0.597, *p* adj. = 0.036), formaldehyde and mannitol-positive *Staphylococcus* spp. (ρ = 0.590, *p* adj. = 0.038), and relative humidity and total bacteria (ρ = 0.579, *p* adj. = 0.043).

Of the 140 tested pairs, 35 were nominally significant (*p* < 0.05), but only 10 survived Benjamini–Hochberg correction. Notably, none of the PM fractions were significantly associated with any microbiological variable after correction, despite their strong contribution to PCA axes. This suggests that while PM concentrations differentiate functional spaces within the zoo, they do not show a direct statistical association with microbial loads at the resolution of this study.

## 3. Materials and Methods

### 3.1. Sampling Strategy of Working Environments in the Zoo

This study was conducted in a zoological garden situated in central Poland. The Municipal Zoological Garden in Łódź LLC. is currently the most modern zoo in Poland. Spanning 16.64 hectares, it is home to over 4500 animals representing 604 species from across the globe. Its largest exhibition pavilion, the Orientarium, showcases endangered species native to Southeast Asia, such as Asian elephants, Sumatran orangutans, false gharials, and Malayan sun bears. The zoo’s remarkable biodiversity and engaging exhibits attract more than one million visitors each year from both Poland and abroad. The sampling sites’ characteristics are listed in Table 1. Measurements at the 24 sampling locations (Figure S1) were conducted sequentially over 2 sampling days during the second half of April 2024 and, consequently, the locations were not monitored simultaneously.

All measurements described in Section 3.1.1, Section 3.1.2, Section 3.1.3 and Section 3.2.1 were conducted at each of the 24 locations listed in Table 1. Additional analyses (safety goggles, settled dust, and cytotoxicity) were conducted at selected locations, as indicated in Table 1.

#### 3.1.1. Microclimate Analysis

A TES-1360 digital humidity meter (TES Electrical Electronic Corp., Taipei, Taiwan) (measurement range: 10–95% ± 3%) was used to evaluate the relative humidity (RH), and a DT-34 digital thermometer (Termoprodukt, Bielawa, Poland) (measurement range: −50 °C to +270 °C ± 0.8 °C) was used to measure temperature (T). The microclimate analysis was performed in eight repetitions at a height of 1.5 m from the ground for each location listed in Table 1.

#### 3.1.2. Airborne Dust Measurement

A portable laser photometer, DustTrak™ DRX Aerosol Monitor 8533 (TSI, Shoreview, MN, USA), was used to evaluate the airborne dust in the working environments of the zoo. The size-segregated fractions were measured as follows: PM_1_, PM_2.5_, RESP (PM_4_), PM_10_, as well as total PM size fractions. The detection range of a portable laser photometer was set from 0.001 to 150 mg/m^3^ for particle sizes of 0.1–15 μm. Measurements were performed at a height of 1.5 m above the floor, using a sampling flow rate of 3 L/min. At each location, PM was monitored for 3 min, with concentrations recorded at 1 s intervals, resulting in 180 measurement points per location. Measurements at the individual locations were conducted sequentially rather than simultaneously.

For each replicate DRX measurement, the coarse aerosol fraction was calculated as the difference between PM_10_ and PM_1_ concentrations (PM_10_ − PM_1_), representing particles with an approximate aerodynamic diameter in the range 1 < d_p_ ≤ 10 μm. Coarse contribution to PM_10_ was calculated from the location-level mean concentrations as 100 × (PM_10_ − PM_1_)/PM_10_.

#### 3.1.3. Airborne Chemical Contamination

A portable gas analyzer, GT5000 Terra Fourier-transform infrared (FTIR) (Gasmet Technologies Oy, Vantaa, Finland), was used to evaluate the chemical contamination in the working environments of the zoo. A gas analyzer allowed a simultaneous analysis of up to 25 gas compounds. The spectrometer specifications were as follows: resolution 8 cm^−1^, Peltier-cooled mercury cadmium telluride (MCT) detector, ZnSe beam splitter, and wave number range 900–4200 cm^−1^. Measurements were performed at a height of 1.5 m from the ground (sample pump flow: 2 L/min) in continuous mode for 10 min (10 scans/s) for each location listed in Table 1.

### 3.2. Microbial Contamination

#### 3.2.1. Determination of Air Microbial Contamination

DUO SAS Super 360 (VWR International Ltd., Milano, Italy), according to CEN EN 13098 [80], was used to evaluate microbiological contamination in the working environments of the zoo. The air samples (100 mL) were collected using the media presented in Table 2. Measurements, with an airflow rate of 100 L/min, were performed in triplicate at a height of 1.5 m from the ground for each location listed in Table 1. Subsequently, the samples were incubated at the designated temperature and time (Table 2) depending on the medium used, followed by colony counting and adjustment based on the sampler manufacturer’s statistical correction table. The results were computed as the arithmetic mean of three independent trials and are expressed in CFU/m^3^ ± standard deviation (SD).

#### 3.2.2. Determination of Surface Microbial Contamination

Samples from 19 different surfaces throughout the facility (Table 1) in two independent repetitions were collected on the days of testing between 8:00 and 13:00 using Hygicult^®^ TPC (Orion Diagnostica Oy, Espoo, Finland) with the Total Plate Count medium. The method was used for comparative screening of culturable surface contamination and was not intended for quantitative exposure assessment. The collected samples were incubated at 30 ± 2 °C for 3–5 days. Next, the colonies were counted, and the results (arithmetic mean of two independent repetitions) are expressed in CFU/cm^2^.

#### 3.2.3. Determination of Protective Goggle Microbial Contamination 

Swabs were taken from the front and back sides of the left and right lenses of protective goggles made from polycarbonate. The goggles’ localization and exposure time are described in Table 3. At each workplace, the goggles were placed on a flat uncovered surface at an approximate height of 1.5 m above the floor. The goggles were positioned lying flat, with the lenses oriented upwards, and remained undisturbed throughout the designated exposure period. Separate pairs of goggles were used for each workplace and exposure time. The reference goggles were stored in the laboratory and were not exposed to the workplace environment. Each workplace–exposure time combination was represented by one independent pair of goggles. Measurements obtained from the individual lens surfaces and duplicate culture determinations were treated as technical subsamples and were not considered independent experimental replicates. After sampling, the swabs were transferred to separate sterile vials containing 5 mL of saline solution (0.85% NaCl). Subsequently, the samples were diluted from 10^−2^ to 10^−6^ in duplicates and plated on TSA and MEA media. The samples were incubated at the designated temperature and time (Table 2) depending on the medium used, followed by colony counting. The results were computed as the arithmetic mean of two trials and expressed in CFU/goggles ± SD.

#### 3.2.4. Determination of Settled Dust Microbial Contamination

Approximately 10 g of settled dust was collected manually from each localization within zoo premises (Table 4) and then transferred to a sterile plastic container prior to analysis. The settled dust samples were mixed, and 0.1 g of each sample was transferred to 9.9 mL of saline solution (0.85% NaCl). Subsequently, the samples were diluted from 10^−2^ to 10^−6^ in duplicates and plated on TSA and MEA media. The samples were incubated at the designated temperature and time (Table 2) depending on the medium used, followed by colony counting. The results were computed as the arithmetic mean of two trials and expressed in CFU/g ± SD.

### 3.3. Determination of Biodiversity

To determine the biodiversity of microorganisms in the air of the zoo, bulk air samples (6000 L) from the employees’ areas (Table 1) and control air samples (200 m from the zoo) were taken. Air was passed through sterile gelatine filters (80 mm, 0.3 µL Sartorius, Germany) using AirPort MD 8 (Sartorius, Göttingen, Germany).

The samples were isolated using the Soil DNA Purification Kit (Eurx, Gdańsk, Poland) according to the manufacturer’s protocol, with one modification—a VWR Mini Bead Mill disrupter (Avantor, Radnor, PA, USA) was used instead of horizontal vortexing. An extraction blank control (sterile, new gelatine filter processed through the complete DNA isolation protocol) was included. DNA concentration in the extraction blank, as measured using the Qubit 4 Fluorometer with the Qubit dsDNA HS Assay (Thermo Fisher Scientific, Waltham, MA, USA), was below the detection limit (“out of range—too low”) confirming negligible carry-over contamination. DNA library preparation and sequencing of 16S rDNA (bacteria) and ITS (fungi) amplicons were performed by genXone (Złotniki, Poland). The V3–V8 regions of the 16S rRNA gene were amplified using the universal primer pair 337F (5′-GACTCCTACGGGAGGCWGCAG-3′) and 1391R (5′-GACGGGCGGTGWGTRCA-3′), while the ITS1-5.8S-ITS2 region was amplified using primers ITS1_F (5′-CTTGGTCATTTAGAGGAAGTAA-3′) and LR3_R (5′-GGTCCGTGTTTCAAGACG-3′) [81,82]. The resulting amplicons were sequenced using nanopore technology with the native barcoding ligation kit V14 (Oxford Nanopore Technologies, ONT, Oxford, UK) protocol on R10.4.1 (ONT) flow cells under default settings.

Sequencing data were processed using two specialized NanoZoo pipelines developed for this study (GitHub repository version 3.22 tomasz-grzyb/nano_zoo), both based on the NaMeCo framework developed by Timur Yergaliyev (GitHub repository timyerg/NaMeco). For bacterial 16S rDNA analysis, reads were first quality-filtered using fastplong (v. 0.2.2) [83] with a Phred q-score threshold ≥ 15, followed by k-mer frequency counting (k = 5). The bacterial pipeline implements a two-stage clustering approach using Uniform Manifold Approximation and Projection (UMAP) and Hierarchical Density-Based Spatial Clustering of Applications with Noise (HDBSCAN) (minimum cluster size = 500, k = 5) [84,85], first clustering per sample and then between samples to identify shared clusters. Consensus sequences were generated using SPOA (v. 4.0.7), followed by read mapping with Minimap2 (v. 2.28) and sequence refinement using Racon (v. 1.4.20) [86,87]. Taxonomic classification was performed using the Genome Taxonomy Database (GTDB) release 220 SSU All Species database with hierarchical identity thresholds [88]. The fungal pipeline consisted of processing and taxonomy modules. The processing module began with the same quality filtering approach, followed by k-mer counting (k = 6) and two-stage clustering with parameters optimized for ITS regions (minimum cluster size = 25). Consensus sequence generation followed the bacterial pipeline approach. The taxonomy module utilized the UNITE + International Nucleotide Sequence Database (INSD) version 10 (Full UNITE + INSD dataset for fungi) [89] database with modified identity thresholds for fungal sequences. Both pipelines produced abundance tables, taxonomic assignments, and quality metrics as outputs.

The obtained data were imported into R (v.4.4.2) using the phyloseq (v. 1.54.0) package and further analyzed and visualized using the following packages: tidyverse (v. 2.0.0), phyloseq (v. 1.54.0), ape (v. 5.8.1), VennDiagram (v. 1.8.2), ggplot2 (v. 4.0.1), and RColorBrewer (v. 1.1.3) [90,91,92,93].

### 3.4. Cell Culture and Cytotoxicity of Settled Dust

Approximately 10 g of settled dust was collected manually from each location listed in Table 4 (except the elephants’ zone—first floor) and transferred to a sterile plastic container prior to cytotoxicity testing. The settled dust samples were mixed, and 0.5 g of each sample was transferred to 5 mL of basal medium for cell culture. The concentration of stock dust solution was calculated as 100 mg/mL. Then, the samples were extracted for 40 min at room temperature (160 rpm) and pH was set to neutral (pH 7.0 ± 0.2). Subsequently, the samples were filtered twice by means of 0.22 μm sterile syringe filters (Membrane Solutions, Kent, WA, USA). The final concentrations of tested extracts were from 0.4 to 100 mg/mL. The cytotoxicity analysis was performed using MTT (3-(4,5-dimethylthiazol-2-yl)-2,5-diphenyltetrazolium bromide) assay, and the human adenocarcinoma lung (alveolar) epithelial adherent cell line A-549 (Cell Line Service GmbH, Eppelheim, Germany) from the 33rd passage. The A-549 cell line is frequently employed for cytotoxicity testing of dust and air pollution since it replicates similar conditions to the ultrafine PM fractions observed in alveoli [4].

The cell culture was performed in Roux-type flasks (Greiner Bio-One GmbH, Frickenhausen, Germany) as a monolayer with Dulbecco’s Modified Eagle Medium (DMEM)/Ham’s F12 (1:1, *v*/*v*) supplemented with 5% fetal bovine serum (FBS), 2 mM glutamine, 25 mM 4-(2-hydroxyethyl)-1-piperazineethanesulfonic acid (HEPES), 100 μg/mL streptomycin, and 100 IU/mL penicillin. Cultures were maintained for 3–5 days at 37 °C in 5% CO_2_. Cells were washed every 2nd day with phosphate-buffered saline (PBS, pH 7.2), and the medium was replaced. At 80% confluence, cells were detached with TrypLE™ Express for 3–5 min at 37 °C, centrifuged (187× *g*, 5 min), and re-suspended in fresh medium. Cells were stained with trypan blue and counted using a hemocytometer. Only cells with ≥80% viability were used for cytotoxicity testing.

For MTT assay, 1 × 10^4^ A-549 cells were seeded per well in a 96-well plate with complete culture medium and incubated for 24 h at 37 °C, 5% CO_2_. The medium was then replaced with 200 µL of each tested extract in 4 replicates; negative controls received the medium only (8 replicates). Cells were incubated for 24 h, followed by the addition of 100 µL MTT (0.5 mg/mL in PBS) for 3 h. Formazan was solubilized with 50 µL dimethyl sulfoxide (DMSO), and absorbance was measured at 550 nm (reference 620 nm) using a microplate reader (TriStar2 LB 942, Berthold Technologies GmbH and Co. KG, Bad Wildbad, Germany). Cell viability (%) was calculated based on the following equation: (sample OD/control OD) × 100; cytotoxicity (%) = 100 − cell viability. Results are presented as mean ± SD. IC_50_ values, representing the concentration causing 50% inhibition of cell survival, were determined from curves according to Organization for Economic Co-operation and Development (OECD) Guidelines [94,95].

### 3.5. Statistical Analysis

All quantitative data were initially checked for normality using the Shapiro–Wilk test and for homogeneity of variance using the Levene’s test. Depending on the distribution properties, either parametric or non-parametric procedures were applied. Microclimatic parameters, airborne dust fractions, gaseous pollutants, and microbial contamination of surfaces and goggles were primarily analyzed using descriptive statistics (mean, standard deviation, minimum, and maximum values). For comparisons between employee and visitor zones, the sampling location was treated as the independent unit of analysis, while repeated instrumental readings were averaged to obtain one location-level value. 

Microclimatic parameters (temperature, relative humidity, and airflow velocity) and PM fractions (PM_1_, PM_2_._5_, RESP, and PM_10_) were compared between 12 employee area and 12 visitor area locations using the Kruskal–Wallis test. Individual gaseous pollutants were also compared between the two zones using the Kruskal–Wallis test. The resulting *p*-values were adjusted separately within each parameter group using the Benjamini–Hochberg procedure. Differences in the overall multivariate gaseous-pollutant profiles between employee and visitor zones were assessed using permutational PERMANOVA, while homogeneity of multivariate dispersion was evaluated using the betadisper procedure. In the case of protective goggles, each workplace–exposure time combination was represented by a single independent pair; therefore, inferential comparisons between exposure times were not performed. Duplicate analytical determinations were treated as technical replicates and were used solely to calculate descriptive summary values.

Airborne microbiological contamination across employees’ and visitors’ sampling sites was compared using the Kruskal–Wallis test, with a significance level set at *p* < 0.05. Results were visualized as boxplots showing median values with minimum–maximum ranges. Cytotoxicity of settled dust was evaluated using one-way ANOVA followed by Tukey’s post hoc test. Statistically homogeneous groups were denoted by letters (a–c), where identical letters indicate no significant difference and different letters represent significant differences (*p* < 0.05). Associations between IC_50_ values and microbiological parameters were not statistically tested because only four location-level IC_50_ estimates were available. In addition, no paired taxonomic sequencing data were obtained for the corresponding settled-dust samples.

All statistical analyses were carried out using Statistica software (v.13.3, StatSoft, Inc., Tulsa, OK, USA), with the significance threshold set at *p* < 0.05.

To explore multivariate relationships between physicochemical and microbiological parameters across sampling sites, principal component analysis (PCA) was performed. Prior to analysis, zero values in microbiological data were replaced with half the limit of detection (LOD/2 = 5 CFU/m^3^), while zeros in chemical data were replaced with half the minimum observed non-zero value per compound. Concentration-based variables (microbial counts, PM fractions, and gaseous pollutants) were log10-transformed, whereas microclimatic parameters (temperature, relative humidity, and airflow velocity) were left untransformed. All variables were subsequently autoscaled (Z-scored). PCA was computed using the prcomp function in R (v.4.5.2). Differences in multivariate environmental profiles between employee and visitor zones were tested using permutational multivariate analysis of variance (PERMANOVA; adonis2 function, 9999 permutations) [96] on Euclidean distances, with homogeneity of multivariate dispersion assessed using the betadisper function [97] (vegan package). The normality of transformed variables was assessed using the Shapiro–Wilk test, which indicated departures from normality for the majority of variables (16 of 27; *p* < 0.05); consequently, non-parametric correlation analysis was applied. Pairwise associations between physicochemical (*n* = 20) and microbiological (*n* = 7) variables were assessed using Spearman’s rank correlation coefficients, with *p*-values adjusted for multiple comparisons using the Benjamini–Hochberg procedure [98]. All analyses were performed in R (v.4.5.2) using the factoextra v. 2.1.0 [99], vegan v. 2.7.2. [100], and Hmisc v. 5.2.5. [101] packages. The significance threshold was set at *p* < 0.05.

## 4. Conclusions

This study presents the first comprehensive investigation of air quality in a zoological garden, encompassing particulate matter, gaseous pollutants, airborne and surface microbiological contamination, microbial accumulation on protective goggles, and the cytotoxicity of settled dust. Concentrations of PM ranged from 0.250 mg/m^3^ for PM_1_ to 0.278 mg/m^3^ for PM_10_, with the highest values recorded in employee workshops, food storage facilities, and visitor-accessible cloakrooms and aquaria. The calculated PM_10_−PM_1_ fraction demonstrated a substantial contribution of coarse particles at several sampling locations, supporting local dust resuspension as an important and potentially modifiable source of airborne particulate matter. Measures limiting resuspension may therefore contribute to reducing coarse-particle concentrations in the zoo environment. Airborne chemical profiles reflected site-specific functions, with ammonia, methane, mercaptans, and volatile organics prevailing in animal and food-related areas, while combustion-related pollutants such as CO and NO_2_ were typical of technical spaces. However, because PM and gaseous contaminants were measured during short, sequential sampling periods, the reported values represent conditions at the time of sampling rather than full-shift or daily exposure and cannot be used to assess compliance with occupational or ambient-air limit values.

Microbiological analyses revealed bacteria as the dominant airborne group, with levels reaching 1.5 × 10^4^ CFU/m^3^ in visitor zones (significantly higher than in employee areas) demonstrating the impact of human circulation on microbial burdens. Fungi, actinomycetes, and opportunistic staphylococci were also identified, while surface contamination remained moderate (mean 4.3 CFU/cm^2^). For the first time, microbial colonization of protective goggles was documented. The highest microbial counts were recorded after 7 days; however, because each workplace–exposure time combination was represented by a single independent pair of goggles, no statistically supported time-dependent trend could be inferred.

Sequencing confirmed diverse microbial communities, dominated by lactic acid bacteria but including opportunistic taxa. Cytotoxicity testing of settled dust using human alveolar epithelial A-549 cells demonstrated dose-dependent toxic effects, with IC_50_ values ranging from 13.1 to 53.5 mg/mL, and the greatest cytotoxicity observed for samples collected from the food storage and workshop areas. However, because only four location-level IC_50_ estimates were available and no paired taxonomic sequencing data were obtained for the corresponding settled-dust samples, a formal correlation analysis between cytotoxicity and microbial load or dominant microbial taxa could not be performed.

When considered together, the data reveal that visitor areas were characterized by higher airborne bacterial concentrations, driven by intensive human traffic and surface contact, while employee areas—though lower in microbial counts—exhibit settled dust collected from selected employee areas showed greater cytotoxicity. However, the relative contributions of the chemical and biological components of settled dust to the observed cytotoxic effects could not be distinguished. This contrast indicates that employee and visitor areas may involve different environmental contamination profiles: employee areas showed more complex chemical–microbial conditions, whereas visitor-accessible areas were characterized by higher airborne bacterial concentrations, which may be particularly relevant in areas frequently used by vulnerable groups such as children. The PCA and correlation analyses further confirm that microclimate and PM gradients differentiate functional zones but do not directly predict microbial loads; rather, operational factors (visitor density, cleaning protocols, ventilation design, and animal species) may contribute to the observed microbial patterns.

The results show that visitor-accessible areas were characterized by higher bacterial concentrations than employee areas, with bacterial concentrations in public areas exceeding those in staff areas by more than three times, requiring a dual risk assessment model that considers both occupational and public exposure, particularly for vulnerable groups such as children. The presence of opportunistic pathogens in visitor areas, combined with the cytotoxic potential of settled dust, indicates the need for priority actions, such as improved ventilation in high-traffic areas, regular microbiological monitoring of public surfaces, informing visitors about age-related risks, and developing a regulatory framework for microbiological air quality in facilities with close interaction between humans and animals. These measures are essential to protect both staff and the increasing number of visitors, especially children who frequently visit zoos.

Future studies could benefit from incorporating measurements of aerosol surface area distribution alongside conventional particle mass metrics. Such an approach may provide additional insight into the physical mechanisms governing microbial transport, deposition, and exposure, thereby contributing to a more comprehensive interpretation of bioaerosol dynamics in indoor zoological environments. An additional limitation of the present study is that the costs and environmental life-cycle impacts of the proposed preventive measures were not assessed. Consequently, the recommended interventions could not be ranked according to their cost-effectiveness or environmental performance. Future studies should evaluate their effectiveness in reducing microbiological contamination alongside their energy demand, maintenance requirements, implementation and operating costs, and carbon footprint.

## Figures and Tables

**Table 1:** Characteristics of sampling sites in employee and visitor zones of the zoo.

Sample ID	Location	Sampling Site	Description	Applied Analyses
Employees’ area				
E1	Veterinary examination room	Lab desk	A specialized room or veterinary clinic where animal health examinations and treatments are administered, and medicine and medical equipment are stored.	D, C, AM, SM
E2	Veterinary bird cage	Wall	A place where sick or healthy birds are kept separately (before or after treatment).	D, C, AM, SM
E3	Employees’ kitchen	Kitchen desk	A resting place for caregivers where they can eat a meal, drink coffee, or simply relax during a break from work.	D, C, AM, SM
E4	Employees’ cloakroom	Wardrobe	A place where caregivers can change into uniforms and store their personal belongings.	D, C, AM, SM
E5	Workshop	Wardrobe and fridge	A place where keepers can repair or create new items of equipment for animals, such as toys, shelters, or elements that enrich their living environment.	D, C, AM, SM, G, SD, Cyt
E6	Food storage	Food box and employee desk	A room intended for storing pet food and supplements, often equipped with refrigerators and freezers for storing foods that require low temperatures.	D, C, AM, SM, G, SD, Cyt
E7	Cold room	Vegetable and meat storages		D, C, AM, SM
E8	Administration office	Countertop and coffee desk	A place where employees can handle administrative matters, work planning, education, and communication with other co-workers.	D, C, AM, SM
E9	Hallway between clean (office workers) and dirty (animal workers) zones	Railing and glass doors	A place that connects the zone of workers engaged with animals and the zone of office workers.	D, C, AM, SM
E10	Elephants’ headquarters	Kitchen desk and catwalk doors	A hallway where caregivers of elephants walk between places within the elephant zone.	D, C, AM, SM, G, SD, Cyt
E11	Zone for scuba divers	Aquarium window and fridge doors	A place where scuba divers work, also a place where isolated fish are kept separately from others.	D, C, AM, SM
E12	Monkey headquarters	Doors	A special place from which keepers can observe animals without disturbing their natural behavior, especially when conducting research on their behavior.	D, C, AM, SM
Visitors’ area				
V1	Reference sampling	Atmospheric air	A place in the parking lot at a distance of 15 m from the main entrance to the zoo.	D, C, AM
V2	Visitors’ cloakroom	Wardrobe and doors	A place where visitors can change and store their personal belongings.	D, C, AM, SM
V3	Elephants’ zone- ground floor	Barrier	A place adjacent to the elephant exposition.	D, C, AM, SM
V4	Macaques zone-ground floor	Window	A place adjacent to the macaques’ exposition.	D, C, AM, SM
V5	Oceanarium—main square	Window	A place adjacent to the aquariums and ocean animals’ exposition.	D, C, AM, SM
V6	Free flight zone-ground floor	Barrier	A place near the crocodiles, orangutan, and free flight zone for birds.	D, C, AM, SD, SM, Cyt
V7	Free flight zone-first floor	N/A		D, C, AM
V8	Macaques’ zone – first floor	N/A	A place adjacent to the macaques’ exposition.	D, C, AM
V9	Elephants’ zone-first floor	N/A	A place adjacent to the elephant exposition.	D, C, AM, SD Cyt
V10	Elephant bathing area	Sculpture and water sample from fountain	A place where visitors can observe the elephants bathing.	D, C, AM, SM
V11	Oceanarium-tunnel	Window	A place adjacent to the aquariums and ocean animals’ exposition.	D, C, AM, SM
V12	Reference sampling	Atmospheric air	A place in front of the main entrance to the building where animals are kept.	D, C, AM

N/A—surface analysis was not performed at this location due to the absence of accessible or suitable sampling material/surfaces; D—airborne dust measurement; C—airborne chemical contamination; AM—airborne microbial contamination; SM—surface microbial contamination; G—protective goggles microbial contamination; SD—settled dust microbial contamination; Cyt—cytotoxicity of settled dust.

**Table 2:** Types of media and incubation parameters according to microorganism type.

Microorganism	Medium	Supplier	Incubation Temperature and Time
Bacteria	Tryptic Soy Agar (TSA) with (0.2%) nystatin	Merck Life Science, Warsaw, Poland	30 ± 2 °C, 48 h
Hemolytic bacteria	Columbia Blood Agar with (0.2%) nystatin	Oxoid, Dardilly Cedex, France	37 ± 2 °C, 24–48 h
Mannitol-positive *Staphylococcus* spp.	Chapman Agar with (0.2%) nystatin	Hi Media Laboratories, Mumbai, India	37 ± 2 °C, 24–48 h
*Pseudomonas fluorescens*	King B medium with (0.2%) nystatin	Hi Media Laboratories, Mumbai, India	30 ± 2 °C, 48 h
Enterobacteriaceae	Violet Red Bile Glucose Agar (VRBG LAB-AGAR) with (0.2%) nystatin	Biocorp, Warsaw, Poland	37 ± 2 °C, 24–48 h
*Actinomycetes*	Pochon’s agar with (0.2%) nystatin	Labomix, Łódź, Poland	25 ± 2 °C, 5–7 days
Fungi	Malt Extract Agar (MEA) with (0.1%) chloramphenicol	Merck Life Science, Warsaw, Poland	25 ± 2 °C, 7 days

**Table 3:** Localization and exposition time of protective goggles in employees’ area.

Sample ID	Workplace	Exposition Time [Days]
E5A	Workshop	7
E5B		3
E5C		1
E6A	Food storage	7
E6B		3
E6C		1
E10A	Elephants’ headquarters	7
E10B		3
E10C		1

**Table 4:** Location of settled dust sampling.

Sample ID	Workplace
Employees’ area	
E5	Workshop
E6	Food storage
E10	Elephants’ headquarters
Visitors’ area	
V6 (A and B)	Free flight zone—ground floor (two separate sampling locations within the area)
V9	Elephants’ zone—first floor

## Data Availability

The original contributions presented in this study are included in the article/Supplementary Material. Further inquiries can be directed to the corresponding authors.

## Supplementary Materials

The following supporting information can be downloaded at https://www.mdpi.com/article/10.3390/ijms27177839/s1.
