# Supplementary material for: Molecular Characterization of the Air Microbiome and Associated Toxicological Hazards in a Zoological Garden
Source: Int J Mol Sci. 2026 Sep 1;27(17):7839. doi: 10.3390/ijms27177839 (PMC13565822; doi:10.3390/ijms27177839)
Supplement: Supplementary file 1 [file ijms-27-07839-s001.zip › ijms-4426609-supplementary.pdf]

Table S1. Microclimatic conditions at the sampling locations; (mean ± standard deviation).

Sampling location	Temperature, ˚C	Relative humidity, %	Air flow velocity, m/s
E1	23.3 ± 0.3	35.1 ± 0.9	0.02 ± 0.01
E2	20.7 ± 0.9	32.5 ± 2.6	0.01 ± 0.02
E3	23.6 ± 0.8	33.6 ± 0.8	0.01 ± 0.00
E4	26.0 ± 0.4	35.6 ± 0.4	0.01 ± 0.01
E5	22.3 ± 1.9	29.0 ± 2.7	0.05 ± 0.02
E6	19.9 ± 0.1	34.3 ± 0.1	0.02 ± 0.02
E7	11.9 ± 3.7	44.1 ± 8.7	0.01 ± 0.01
E8	20.5 ± 2.6	30.9 ± 5.7	0.09 ± 0.02
E9	23.2 ± 0.7	23.5 ± 1.1	0.13 ± 0.06
E10	23.8 ± 0.9	23.6 ± 2.1	0.13 ± 0.02
E11	25.2 ± 0.1	36.8 ± 0.7	0.13 ± 0.06
E12	25.0 ± 1.0	27.9 ± 3.1	0.01 ± 0.01
V1	18.3 ± 0.2	88.9 ± 1.2	0.07 ± 0.02
V2	23.9 ± 0.6	60.9 ± 4.5	0.02 ± 0.01
V3	24.1 ± 0.5	56.9 ± 1.4	0.17 ± 0.07
V4	22.5 ± 0.4	59.9 ± 1.8	0.08 ± 0.05
V5	25.3 ± 0.5	53.6 ± 0.7	0.12 ± 0.05
V6	24.9 ± 0.4	54.2 ± 1.2	0.26 ± 0.03
V7	24.8 ± 0.8	54.2 ± 2.4	0.08 ± 0.04
V8	23.4 ± 0.4	56.7 ± 1.9	0.14 ± 0.06
V9	26.3 ± 0.4	50.1 ± 1.7	0.23 ± 0.06
V10	24.4 ± 1.4	54.4 ± 3.2	0.06 ± 0.02
V11	23.5 ± 0.3	56.9 ± 0.6	0.10 ± 0.11
V12	20.6 ± 0.6	63.9 ± 0.9	0.16 ± 0.07

Table S2. Data set on airborne microbial concentrations for all tested sampling locations

Type of microorganism	Employees' area			Visitors' area		
	Location	Mean	Standard deviation	Location	Mean	Standard deviation
Fungi	E1	1.47E+02	9.07E+01	V1	8.47E+02	1.34E+02
	E2	1.30E+02	5.20E+01	V2	3.10E+02	2.00E+01
	E3	1.02E+03	3.00E+02	V3	1.53E+02	4.51E+01
	E4	1.44E+03	2.53E+02	V4	2.50E+02	2.65E+01
	E5	9.33E+01	1.53E+01	V5	1.37E+02	5.77E+00
	E6	1.63E+02	2.52E+01	V6	6.90E+02	8.72E+01
	E7	3.37E+02	3.06E+02	V7	2.19E+03	0.00E+00
	E8	2.00E+01	1.73E+01	V8	2.87E+02	6.66E+01
	E9	7.33E+01	1.53E+01	V9	3.57E+02	7.51E+01
	E10	6.83E+02	1.16E+02	V10	1.90E+02	7.21E+01
	E11	1.63E+02	5.69E+01	V11	6.37E+02	7.22E+02
	E12	7.00E+01	3.46E+01	V12	4.37E+02	5.51E+01
Bacteria	E1	3.60E+02	8.66E+01	V1	3.23E+03	4.04E+02
	E2	2.27E+02	6.66E+01	V2	2.19E+03	0.00E+00
	E3	1.87E+03	1.72E+02	V3	4.03E+03	3.18E+03
	E4	2.73E+03	3.82E+02	V4	1.19E+04	8.44E+03

	E5	1.79E+03	3.72E+02	V5	1.54E+04	3.99E+03
	E6	3.00E+03	0.00E+00	V6	2.19E+03	0.00E+00
	E7	2.43E+03	9.81E+02	V7	2.19E+03	0.00E+00
	E8	1.07E+02	4.04E+01	V8	2.19E+03	0.00E+00
	E9	9.53E+02	5.51E+01	V9	2.19E+03	0.00E+00
	E10	3.00E+03	0.00E+00	V10	2.19E+03	0.00E+00
	E11	1.80E+02	1.00E+01	V11	2.19E+03	0.00E+00
	E12	1.40E+02	7.00E+01	V12	3.86E+03	2.89E+03
Actinomycetes	E1	2.33E+01	1.53E+01	V1	1.00E+01	1.00E+01
	E2	0.00E+00	0.00E+00	V2	1.47E+02	2.08E+01
	E3	1.27E+02	5.51E+01	V3	1.93E+02	1.22E+02
	E4	1.57E+02	1.07E+02	V4	3.73E+02	2.31E+01
	E5	3.33E+00	5.77E+00	V5	2.53E+02	3.79E+01
	E6	2.67E+01	2.52E+01	V6	2.67E+01	1.53E+01
	E7	7.00E+01	8.66E+01	V7	4.00E+01	4.58E+01
	E8	3.67E+01	3.79E+01	V8	2.97E+02	9.07E+01
	E9	1.07E+02	2.08E+01	V9	3.17E+02	7.23E+01
	E10	1.09E+03	1.66E+03	V10	3.37E+02	8.08E+01
	E11	4.67E+01	1.53E+01	V11	5.17E+02	6.66E+01
	E12	3.00E+01	1.73E+01	V12	9.00E+01	1.00E+01
Mannitol-positive *Staphylococcus* spp.	E1	4.00E+01	1.73E+01	V1	1.33E+01	1.53E+01
	E2	1.67E+01	5.77E+00	V2	3.30E+02	2.65E+01
	E3	2.00E+01	1.73E+01	V3	1.10E+02	2.00E+01
	E4	6.33E+01	3.06E+01	V4	1.57E+02	7.09E+01
	E5	4.33E+01	3.21E+01	V5	1.57E+02	3.06E+01
	E6	2.33E+01	1.53E+01	V6	7.17E+02	9.29E+01
	E7	8.33E+01	3.51E+01	V7	7.50E+02	4.58E+01
	E8	3.33E+00	5.77E+00	V8	3.97E+02	9.07E+01
	E9	2.00E+01	1.00E+01	V9	3.73E+02	2.24E+02
	E10	3.73E+02	1.08E+02	V10	3.13E+02	4.51E+01
	E11	3.33E+00	5.77E+00	V11	1.40E+02	1.73E+01
	E12	6.67E+00	5.77E+00	V12	5.00E+01	2.65E+01
Haemolytic Staphylococcus	E1	5.67E+01	1.53E+01	V1	3.33E+00	5.77E+00
	E2	1.33E+01	1.15E+01	V2	2.23E+02	5.51E+01
	E3	9.33E+01	5.77E+00	V3	1.30E+02	4.58E+01
	E4	1.03E+02	2.52E+01	V4	1.50E+02	2.65E+01
	E5	1.93E+02	3.79E+01	V5	1.53E+02	8.08E+01
	E6	4.00E+01	1.00E+01	V6	2.90E+02	7.81E+01
	E7	4.67E+01	3.06E+01	V7	2.73E+02	4.73E+01
	E8	2.67E+01	5.77E+00	V8	1.90E+02	3.61E+01
	E9	1.67E+01	1.15E+01	V9	3.63E+02	5.03E+01
	E10	1.70E+02	8.19E+01	V10	9.33E+01	7.77E+01
	E11	1.00E+01	0.00E+00	V11	2.50E+02	1.91E+02
	E12	2.67E+01	5.77E+00	V12	2.00E+01	1.73E+01
Pseudomonas fluorescens	E1	0.00E+00	0.00E+00	V1	3.33E+00	5.77E+00
	E2	0.00E+00	0.00E+00	V2	0.00E+00	0.00E+00
	E3	0.00E+00	0.00E+00	V3	3.33E+00	5.77E+00
	E4	3.33E+00	5.77E+00	V4	3.33E+00	5.77E+00

	E5	0.00E+00	0.00E+00	V5	0.00E+00	0.00E+00
	E6	3.33E+00	5.77E+00	V6	6.67E+00	5.77E+00
	E7	3.33E+00	5.77E+00	V7	6.67E+00	1.15E+01
	E8	0.00E+00	0.00E+00	V8	3.33E+00	5.77E+00
	E9	0.00E+00	0.00E+00	V9	0.00E+00	0.00E+00
	E10	6.67E+00	1.15E+01	V10	1.67E+01	2.89E+01
	E11	0.00E+00	0.00E+00	V11	3.33E+00	5.77E+00
	E12	0.00E+00	0.00E+00	V12	0.00E+00	0.00E+00
Enterobacteriaceae	E1	0.00E+00	0.00E+00	V1	1.50E+01	5.00E+00
	E2	0.00E+00	0.00E+00	V2	5.00E+00	5.00E+00
	E3	0.00E+00	0.00E+00	V3	0.00E+00	0.00E+00
	E4	0.00E+00	0.00E+00	V4	0.00E+00	0.00E+00
	E5	3.33E+00	5.77E+00	V5	2.50E+01	1.50E+01
	E6	3.33E+00	5.77E+00	V6	1.50E+01	5.00E+00
	E7	3.33E+00	5.77E+00	V7	2.50E+01	5.00E+00
	E8	0.00E+00	0.00E+00	V8	1.00E+01	1.00E+01
	E9	0.00E+00	0.00E+00	V9	1.50E+01	1.50E+01
	E10	0.00E+00	0.00E+00	V10	5.00E+00	5.00E+00
	E11	0.00E+00	0.00E+00	V11	1.50E+01	1.50E+01
	E12	0.00E+00	0.00E+00	V12	5.00E+00	5,.0E+00

Table S3. Calculated coarse aerosol fraction for all tested sampling locations.

Sampling location	PM_1, mg/m^3 (mean ± SD)	PM_{10}, mg/m^3 (mean ± SD)	PM_{10}–PM_1, mg/m^3 (mean ± SD)	Coarse contribution to PM_{10}, %
E1	0.01960 ± 0.00089	0.02400 ± 0.00122	0.00440 ± 0.00055	18.3
E2	0.01680 ± 0.00045	0.01880 ± 0.00045	0.00200 ± 0.00000	10.6
E3	0.01480 ± 0.00110	0.01700 ± 0.00141	0.00220 ± 0.00045	12.9
E4	0.01175 ± 0.00450	0.01425 ± 0.00556	0.00250 ± 0.00129	17.5
E5	0.24975 ± 0.15782	0.27750 ± 0.17222	0.02775 ± 0.01910	10.0
E6	0.03650 ± 0.01678	0.05825 ± 0.03037	0.02175 ± 0.01360	37.3
E7	0.02075 ± 0.00171	0.02550 ± 0.00208	0.00475 ± 0.00050	18.6
E8	0.00025 ± 0.00050	0.00025 ± 0.00050	0.00000 ± 0.00000	0.0
E9	0.00275 ± 0.00150	0.00525 ± 0.00222	0.00250 ± 0.00100	47.6
E10	0.00525 ± 0.00250	0.00725 ± 0.00450	0.00200 ± 0.00200	27.6
E11	0.06725 ± 0.01735	0.11275 ± 0.02902	0.04550 ± 0.01173	40.4
E12	0.00775 ± 0.00050	0.00925 ± 0.00050	0.00150 ± 0.00058	16.2
V1	0.02250 ± 0.00332	0.02325 ± 0.00340	0.00075 ± 0.00050	3.2
V2	0.09800 ± 0.03295	0.16450 ± 0.05345	0.06650 ± 0.02053	40.4
V3	0.02525 ± 0.00866	0.03850 ± 0.01580	0.01325 ± 0.00718	34.4
V4	0.02125 ± 0.00465	0.03100 ± 0.00668	0.00975 ± 0.00206	31.5
V5	0.04750 ± 0.00839	0.07975 ± 0.01654	0.03225 ± 0.00822	40.4
V6	0.03150 ± 0.00551	0.05125 ± 0.00877	0.01975 ± 0.00330	38.5
V7	0.02625 ± 0.00591	0.04350 ± 0.00915	0.01725 ± 0.00330	39.7
V8	0.03000 ± 0.00775	0.04450 ± 0.01063	0.01450 ± 0.00300	32.6
V9	0.04350 ± 0.01292	0.06825 ± 0.02220	0.02475 ± 0.00932	36.3
V10	0.02600 ± 0.00424	0.03900 ± 0.00716	0.01300 ± 0.00294	33.3
V11	0.02925 ± 0.00287	0.04700 ± 0.00356	0.01775 ± 0.00096	37.8
V12	0.02700 ± 0.00365	0.03450 ± 0.00661	0.00750 ± 0.00300	21.7

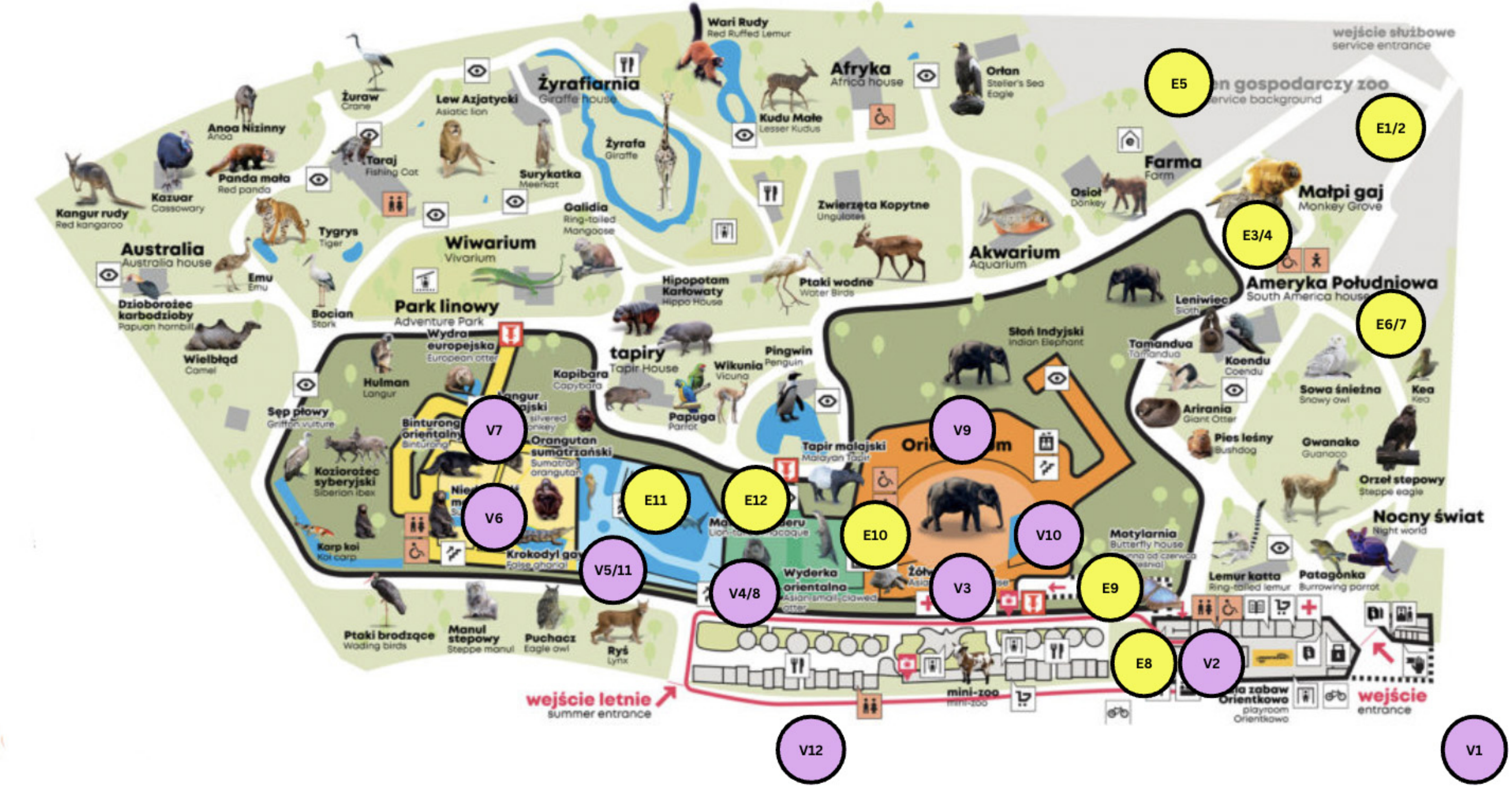

Figure S1. Schematic map of the zoo showing the locations of all air sampling sites, the different animal habitats, and the connecting corridors. Sampling locations are labeled according to the identifiers used in Table S1. Yellow indicates sampling locations in staff-only areas, whereas pink indicates sampling locations in visitor-accessible areas.
